# Genomic profiling of T-cell activation suggests increased sensitivity of memory T cells to CD28 costimulation

**DOI:** 10.1038/s41435-020-00118-0

**Published:** 2020-11-23

**Authors:** Dafni A. Glinos, Blagoje Soskic, Cayman Williams, Alan Kennedy, Luke Jostins, David M. Sansom, Gosia Trynka

**Affiliations:** 1 Wellcome Sanger Institute, Wellcome Genome Campus, Hinxton, CB10 1SA UK; 2 New York Genome Center, New York, NY 10013 USA; 3 Open Targets, Wellcome Genome Campus, Hinxton, CB10 1SA UK; 4 UCL Institute of Immunity and Transplantation, Royal Free Hospital, London, NW3 2PF UK; 5 Kennedy Institute of Rheumatology, University of Oxford, Roosevelt Drive, Oxford, OX3 7FY UK; 6 Big Data Institute, University of Oxford, Roosevelt Drive, Oxford, OX3 7FZ UK; 7 Christ Church, St. Aldates, Oxford, OX1 1DP UK

**Keywords:** Gene expression profiling, Immunogenetics

## Abstract

T-cell activation is a critical driver of immune responses. The CD28 costimulation is an essential regulator of CD4 T-cell responses, however, its relative importance in naive and memory T cells is not fully understood. Using different model systems, we observe that human memory T cells are more sensitive to CD28 costimulation than naive T cells. To deconvolute how the T-cell receptor (TCR) and CD28 orchestrate activation of human T cells, we stimulate cells using varying intensities of TCR and CD28 and profiled gene expression. We show that genes involved in cell cycle progression and division are CD28-driven in memory cells, but under TCR control in naive cells. We further demonstrate that T-helper differentiation and cytokine expression are controlled by CD28. Using chromatin accessibility profiling, we observe that AP1 transcriptional regulation is enriched when both TCR and CD28 are engaged, whereas open chromatin near CD28-sensitive genes is enriched for NF-kB motifs. Lastly, we show that CD28-sensitive genes are enriched in GWAS regions associated with immune diseases, implicating a role for CD28 in disease development. Our study provides important insights into the differential role of costimulation in naive and memory T-cell responses and disease susceptibility.

## Introduction

The ability of T cells to respond to pathogens whilst remaining tolerant to host antigens is critical for human health. Excessive activation of memory T cells is a hallmark of many common complex immune diseases, such as autoimmune arthritis and systemic lupus erythematosus (SLE) [1, 2]. Genome-wide association studies (GWAS) have mapped numerous risk variants to loci encoding genes regulating T-cell stimulatory pathways, including the *CD28*, *CTLA4* and *ICOS* genes located at 2q33.2 [3–6]. While the exact effects of the associated variants are unknown, their mapping to the non-coding regions of the genome suggests effects on gene expression regulation [7, 8]. This implies that immune disease GWAS variants could act through the modulation of the activity of costimulatory pathways. Two recent studies have shown that GWAS variants associated with immune diseases are enriched in chromatin regions active upon T-cell stimulation [9, 10]. The strongest SNP enrichment signal was observed in early activation of memory CD4 T cells [10], indicating that associated variants converge on the regulation of pathways in early events of cell activation, prior to the first cell division. With this genetic anchor to memory T-cell activation, building a better understanding of pathways underlying T-cell activation processes could point towards novel drug targets.

T-cell stimulation initially occurs in secondary lymphoid tissues where T cells interact with professional antigen-presenting cells (APCs). Here, two coordinated signals are delivered: the first via T-cell receptor (TCR) recognising antigen bound to MHC molecules and the second provided by APCs via upregulation of costimulatory ligands. In this regard, CD28 is the main costimulatory receptor expressed by T cells that interacts with CD80 and CD86 ligands on APCs. The coordination of TCR and CD28 signals is essential for T-cell activation, proliferation, differentiation and survival, making the CD28 pathway a key checkpoint for controlling T-cell responses [11, 12]. The level of CD28 costimulation varies considerably in different immunological settings. For example, the presence of regulatory T cells (Tregs) expressing CTLA-4 can degrade CD80 and CD86 ligands [13] influencing CD28 costimulation. Indeed, deficiency in expression of CTLA-4 is associated with the development of profound autoimmune diseases [14–17] due to increased CD28 signalling [18, 19]. Modulating T-cell activation by targeting the CD28 pathway with CTLA4-Ig has also been a successful approach in treating complex immune diseases [20].

Here we combine immunogenomic approaches to study the requirements of TCR and CD28 in the activation of CD45RA^+^ and CD45RA^−^ human CD4 T-cell subsets by stimulating cells with varying intensities of TCR and CD28. First, we used a combination of cellular models to determine whether these T-cell subsets responded in a similar way to CD28 costimulation. Marked differences were observed in control of cell division, which we found to be controlled by CD28 in CD45RA^−^ cells whilst predominantly driven by TCR in CD45RA^+^ cells. We then used gene expression and chromatin activity profiling to map transcriptional processes resulting from stimulation of these receptors in isolation and together. We show that the major effector functions, such as T-helper (Th) differentiation, expression of chemokine receptors and cytokines, were all strongly influenced by CD28 in both cell subsets. Finally, we show that a proportion of variants associated with common immune diseases is enriched in CD28-sensitive genes, pointing towards the important role of this costimulatory pathway in disease pathogenesis. We provide a website to examine the cell type and stimulation-specific gene expression www.sanger.ac.uk/science/tools/costimulation/costimulation.

## Results

### CD28 drives proliferation in CD45RA^−^ memory T cells

**Fig. 1:**
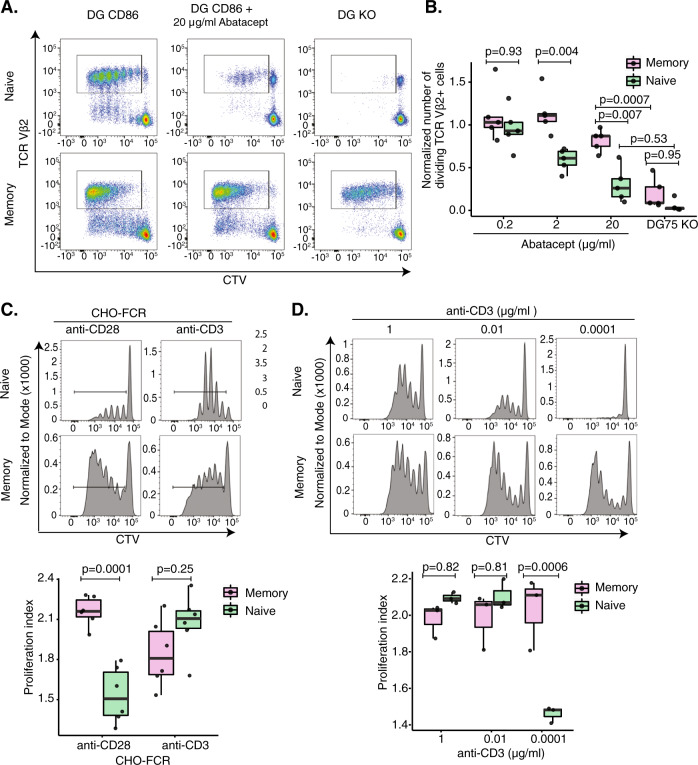
CD28 stimulation drives proliferation of memory T cells. **A**, **B** Purified CD4^+^CD25^−^ memory and naive T cells were CTV stained and stimulated for 5 days in the presence of CD86 transduced DG75 B cells, TSST-1 superantigen and abatacept at the indicated concentrations. The proliferation of Vβ2+ T cells was determined via flow cytometry and the number of cells within the ‘Dividing Vβ2+’ gate calculated using Accucheck counting beads and FLOWJO proliferation software. **B** Boxplots represent the number of cells within the ‘Dividing Vβ2+’ gate relative to controls (no abatacept) for each dose of TSST-1. **C** Purified CTV stained CD4^+^CD25^−^ memory and naive were stimulated with anti-CD28 or anti-CD3 in the presence of CHO-FcR transfectants. The proliferation of memory and naive T cells was measured by flow cytometry five days following stimulation. The proliferation index and precursor frequency were determined using the proliferation calculator of FLOWJO software. **D** Purified CTV stained CD4^+^CD25^−^ memory and naive were stimulated in the presence of CD86 transduced DG75 cells at a T:DG ratio of 1:1 and indicated concentrations of soluble anti-CD3 for 5 days. Proliferation was determined by flow cytometry. **B**–**D** Significance was calculated using two-way ANOVA and group means were compared using Tukey’s honest significant difference test.

To study the requirement for CD28 costimulation in different subsets of human T cells, we used a B cell line (DG75) expressing CD86 (a major CD28 ligand in vivo [21]) to engage CD28 and the superantigen TSST-1 as a ligand for the TCR. For the purposes of this study, we define naive cells as CD45RA^+^ and memory cells as CD45RA^−^, accepting that they are imperfect markers of these cell subsets. We initially modulated CD28 costimulation by titrating abatacept (CTLA4-Ig), a drug used in treating immune diseases (e.g. rheumatoid arthritis) that reduces T-cell activation by blocking costimulation. We compared the proliferative responses of naive and memory T cells by gating on the high affinity responding T cells (TCR Vβ2^+^) to ensure strong recognition of TSST. These data revealed that both memory and naive T cells responded well in the absence of abatacept, and that naive cells were sensitive to abatacept inhibition above 2 μg/ml (Fig. 1A, B and Supplementary Fig. S1A). In contrast, memory cells only showed inhibition at 10-fold higher concentrations of abatacept and more extended cell division compared to naive cells. These data are in line with previous concepts, that memory T cells are less dependent on costimulation compared to naive T cells, continuing to proliferate when CD28 engagement is blocked. However, the data can also be interpreted in a different way, by accepting that CD86 is not completely blocked by abatacept and therefore residual levels of free CD86 might be sufficient to support proliferation in memory but not naive T cells. Viewing the data in this way it can be argued that CD45RA^−^ cells are in fact more sensitive to low levels of residual CD28 engagement. To address this, we also tested responses with DG75 cells where CD86 was deleted using CRISPR. Complete loss of CD28 ligands caused substantial further inhibition particularly in memory cells (Fig. 1B) indicating that the abatacept hypothesis might be true. These data were consistent across several different donors and supported a model in which CD45RA^−^ memory T cells appear to be more sensitive to CD28 signals such that blockade by abatacept is unable to completely disrupt CD28 costimulation.

Since previous studies have shown that CD28 superagonist antibodies can trigger memory T-cell responses in vivo [22, 23], we also investigated how crosslinking CD3 and CD28 antibodies affected naive and memory T-cell responses. Using CHO cells expressing an Fc-gamma Receptor (FcR) to provide antibody crosslinking, we assessed the proliferation of T cells to CD3 or CD28 stimulation alone (Fig. 1C and Supplementary Fig. S1B). Five days following stimulation, naive T cells had proliferated strongly to anti-CD3 alone, committing the majority of cells into division, whereas the response of memory T cells was more variable. In contrast, CD28 crosslinking resulted in a robust response in memory T cells which was not seen in naive T cells (*p*-value = 0.0001). Furthermore, the majority of proliferating memory cells after CD28 stimulation proceeded to later divisions (Fig. 1C), which was less marked in CD3-stimulated memory CD4^+^ T cells. Together these data supported the concept that memory cells can utilise CD28 costimulation more effectively than naive cells and that conversely naive cells were more responsive to TCR stimulation. Together these data imply a switch in the dominance of these costimulatory receptors in the transition from CD45RA^+^ to CD45RA^−^ phenotype.

We next performed typical costimulation experiments where both TCR and CD28 signals were present, fixing CD28 costimulation whilst titrating the strength of the TCR signal via anti-CD3 (Fig. 1D and Supplementary Fig. S1C). This revealed several findings consistent with the above observations. Firstly, titration of TCR stimulus affected naive T cells more than memory, and at the lowest dose of CD3 T-cell proliferation was abrogated in naive cells but not in memory. We hypothesised that this was because memory T cells are supported by more effective CD28 costimulation, whereas naive cells are more dependent on the TCR and therefore susceptible to its loss. Secondly, we observed that at lower doses of anti-CD3 (Fig. 1D), memory T cells also showed more extensive division consistent with a CD28-dominated response and similar to that seen with CD28 signalling alone (Fig. 1C).

Taken together, using several different approaches, the above data suggest that naive and memory T-cell activation is differentially influenced by the dominance of TCR and CD28 signalling. Whilst both naive and memory T cells utilise costimulation, memory T cells appear more sensitive to CD28 than naive T cells, hence their weaker inhibition by abatacept and their more robust response to direct CD28 crosslinking. Additionally, CD28 costimulation in memory T cells results in enhanced proliferation allowing T cells to progress more effectively into later divisions. In contrast, naive T cells predominantly utilise TCR signalling to drive proliferation and are therefore more sensitive to the loss of weak CD28 signals during abatacept inhibition and to depletion of TCR signals.

### Naive and memory T cells operate different gene expression programmes upon activation

In order to further understand the impact of differential utilisation of CD28 signalling by naive and memory cells, we profiled gene expression across different stimulatory conditions. By titrating anti-CD3 antibody and CD86 expressing transfectants, we generated four costimulatory conditions (lowTCR + lowCD28, lowTCR + highCD28, highTCR + lowCD28, highTCR + highCD28) as well as independent signals from TCR (highTCR) and CD28 (highCD28) which were delivered by crosslinking anti-CD3 and anti-CD28 as above (Fig. 1B and Supplementary Fig. S2A, B).

**Fig. 2:**
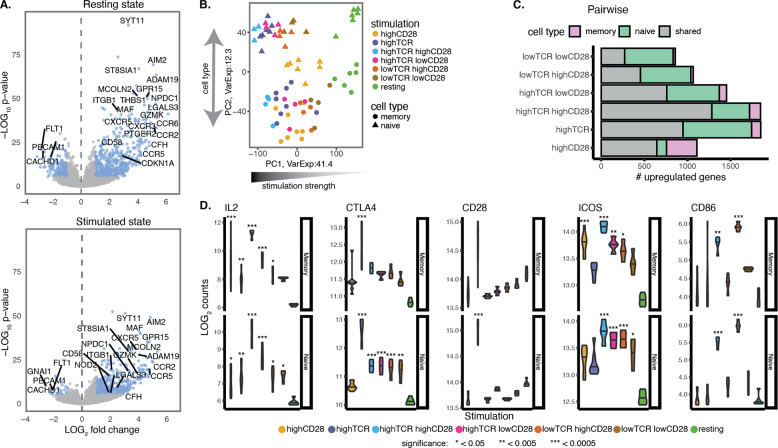
Naive and memory T cells respond differently to TCR and CD28 stimulation. **A** Volcano plot of differential gene expression test between resting naive cells and resting memory cells and stimulated naive cells and resting memory cells. Genes coloured in blue correspond to differentially expressed genes with log2 fold-change >1 and FDR < 5%. Labelled is DEG with the lowest *p*-values. **B** Principal component analysis using the expression of all genes. The first two components explain collectively 53.7% of the observed variability and correlate with stimulation strength and cell type. Each dot corresponds to an individual sample, coloured by stimulation and shaped according to the cell type. **C** Number of upregulated genes upon stimulation defined by pairwise differential expression test between stimulated cells and resting cells (fold-change ≥ 2 and FDR ≤ 0.05). **D** Examples of genes upregulated in response to stimulation. Plots show the read counts across the different stimulatory conditions. *p*-values were calculated using the DESeq2 Wald-test by comparing stimulations to the resting state.

Following 16 h stimulation, we sorted activated CD25^+^CD45RA^+^ naive and CD45RA^−^ memory subsets (see ‘Materials and methods’ section). By comparing the gene expression profiles of the two cell types in the resting and stimulated states, we observed gene expression signatures in each subset, concordant with published cell markers [24] (Fig. 2A and Supplementary Table 1). Among the 294 genes differentially upregulated in memory cells, we found genes involved in the migration of T cells (e.g. chemokine receptors *CXCR3*, *CCR6* and *GPR1*, cell adhesion molecule *CD58* (LFA-3) and β1 integrins), intracellular signalling (phosphatases, calcium signalling molecules, e.g. *SYT11*, *ITPRIPL1*, and kinases, e.g. *CDKN1A*), memory T-cell survival and homoeostasis (cytokine receptors (e.g. *IL1R1*, *IL2RB*, *IL12RB2* and *IL18RAP*), lectins and *FAS*) and transcription factors affecting T-cell differentiation (e.g. *MAF*, *TBX21*, *RORC*, *BHLHE40* and *PRDM1*). *PECAM1* (CD31), a well-known marker of a subset of naive T cells [25], was among the 33 genes differentially upregulated in naive cells. This confirmed that our sorting strategy based on the CD45RA marker successfully captured populations with the predominant phenotype of naive and memory CD4^+^ T cells.

As expected, across different stimulatory conditions, we observed a variable proportion of activated T cells (11–78% of all cells) (Supplementary Fig. S2C). However, by sorting only CD25^+^ T cells, we ensured that the measured gene expression reflected cell activation states induced by different signal intensities, while not being confounded by the variable percentage of activated cells. To assess the global differences in the transcriptome of cells from the different stimulatory conditions, we applied principal component analysis (PCA) and observed that PC1 reflected cell stimulation (explaining 41.4% of the variability) while PC2 corresponded to the cell type (explaining 12.3% of the variability; Fig. 2B). Indeed, when looking directly into the variability explained by each factor, the majority of the gene expression variance was explained by stimulation (47%) and by the differences in cell type (10%) (Supplementary Fig. S2D and ‘Materials and methods’ section). The separation of naive and memory T cells by PC2 confirmed clear differences in transcriptional responses between naive and memory T cells indicating that despite being an imperfect marker of naive and memory cells CD45RA was sufficient to separate these subsets at a global gene expression level. The PCA also captured a gradient of stimulation intensity in both cell types, with strong costimulation conditions separated furthest from unstimulated cells on PC1 and intermediate intensity of stimuli mapping in between. Surprisingly, strong CD28 alone (FcR-anti-CD28) was amongst the lower responding conditions in naive cells yet it clustered with the more highly stimulated conditions in memory cells. Thus, the global analysis of transcriptional programmes also captured the functional differences between naive and memory T cells observed previously.

To understand subset-specific responses induced by the two stimuli, we compared gene expression profiles between resting and stimulated naive and memory T cells (false discovery rate (FDR) ≤ 0.05 and fold-change ≥ 2; Fig. 2C and Supplementary Table S2). As expected, the majority of the upregulated genes were shared between the two cell types, however, naive cells displayed a larger number of differentially upregulated genes (DEGs) (mean = 1243) than memory cells (mean = 847; paired *t*-test *p*-value = 0.034). This likely reflects the larger changes in gene expression levels resulting from transitioning to activation from a deeper quiescent state in naive cells. The exception to this was CD28 stimulation alone, which upregulated more genes in memory cells. Differential expression analysis between naive and memory cells in the unstimulated state revealed a larger number of genes expressed highly in memory cells as expected (Fig. 2A).

To assess the sensitivity of gene expression regulation across the different stimuli, we investigated the expression profiles of selected genes. We observed that gene expression was sensitive to different levels of stimuli and varied between the two cell types. For example, *IL2* expression, which is regulated by costimulation [26], was upregulated in all conditions except memory cells stimulated with low doses of combined CD28 and TCR costimulation. Interestingly, *IL2* was upregulated in response to high CD28 alone in memory cells but not in naive cells (Fig. 2D). We also examined the gene expression profiles of CD28, CTLA4 and ICOS encoded within a single 260 kb locus. We observed that despite their close proximity each gene had a unique mode of expression. For example, CD28 was upregulated significantly only in naive cells in response to high amounts of TCR alone whereas *CTLA4* was significantly upregulated with high TCR stimulation, but not by CD28 stimulation. In addition, *CTLA4* was upregulated by activation generally in naive cells, with strong TCR alone being the most effective. In contrast, *ICOS* expression showed a general requirement for CD28 costimulation resulting in significant upregulation in both naive and memory cells. Finally, we observed that CD86 expression was responsive only in the presence of strong CD28 engagement, but in the presence of TCR stimulation. Together these observations indicate that gene expression in naive and memory cells shows different patterns of TCR and CD28 responsiveness, which are sensitive to both the specific stimulus and its intensity.

### Titration of TCR and CD28 identifies genes sensitive to specific signals

**Fig. 3:**
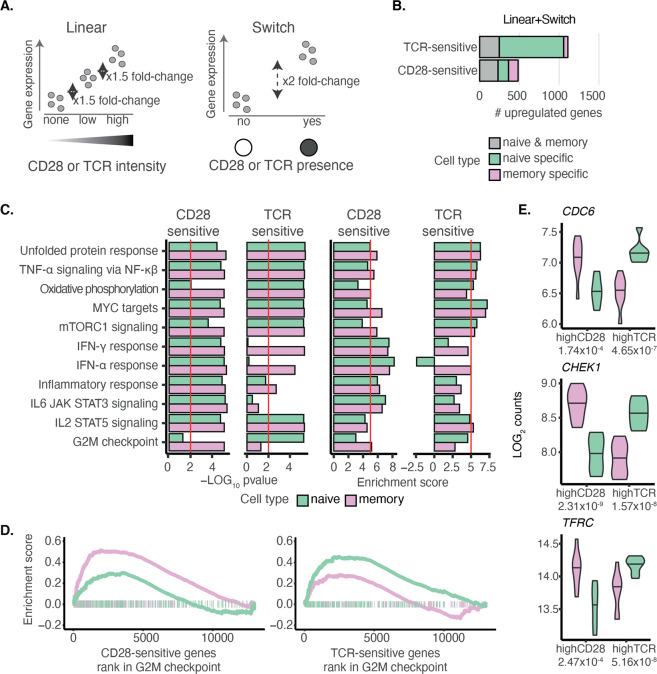
TCR and CD28 titration identifies genes sensitive to each signal. **A** In the linear model, we required a linear increase of gene expression along with stimulus intensity (incremental fold-change ≥ 1.5 in gene expression), separately evaluating naive and memory cells. Genes that did not follow the linear model were tested for the switch model. Here, we assumed an ‘on-and-off’ mode of expression where a gene is significantly upregulated (fold-change ≥ 2) in response to the presence of either CD28 or TCR. In both of these models, we used all seven conditions, e.g. when testing for CD28-sensitive genes, we grouped the TCR alone stimulation with the resting since neither received CD28 signal. A gene was classified in one of the two categories without overlap and prioritised for the linear model. **B** Comparison of the number of genes in naive and in memory cells that are TCR or CD28 sensitive. **C** Hallmark pathways enriched for TCR-sensitive and CD28-sensitive genes in naive and memory cells using fgsea. **D** Pathway enrichment plot for G2M checkpoint. **E** Gene expression profiles of selected switcher genes, genes that change stimulus sensitivity between the two cell types. The number below the stimulus name represents the significance of the difference between naive and memory T cells derived using *t*-test.

Based on the above observations, we sought to comprehensively assess the role of each stimulatory signal in gene upregulation in naive and memory T cells. To classify genes as either CD28^−^ or TCR-sensitive, we used two classification models of gene expression (linear and switch). The linear model reflected genes that changed their expression in response to stimulation intensity, whereas the switch model reflected a digital on/off state of gene expression (Fig. 3A). In both cell types, we were able to assign a unique stimulus sensitivity to 1566 genes, meaning that the expression of these genes was more sensitive to either TCR or CD28 intensity. We observed that the majority of stimulus-sensitive genes (84%) followed the linear model (Supplementary Fig. S3A), suggesting that the expression of a gene is tunable by the signal intensity, rather than by the signal’s presence or absence.

We next assessed whether naive and memory cells differed in sensitivity to the two stimuli and found that the proportions of genes associated to each stimulus differed in the two cell types (chi-square *p*-value = 1.6 × 10^−94^). We observed that the majority of genes in naive T cells were TCR-sensitive (1056; Fig. 3B and Supplementary Table S3), whereas a smaller number of genes was CD28-sensitive (*n* = 360). However, in memory cells, we observed that a larger proportion of genes were CD28-sensitive (*n* = 351) than TCR-sensitive (*n* = 299). As such, we concluded that TCR-sensitive genes and CD28-sensitive genes were unevenly distributed between naive and memory cells, with a shift towards naive cells for TCR-sensitive genes (Fisher’s exact test *p*-value = 1.33 × 10^−82^) and a shift towards memory cells for CD28-sensitive genes (Fisher’s exact test *p*-value = 2.15 × 10^−4^). Based on pairwise comparisons within cell types and across the six conditions against the resting state, we defined a group of genes that were upregulated upon stimulation. Among this group, we observed that the expression of 1224 genes in naive cells (55%) and only 489 genes in memory cells (29%) was sensitive to a single stimulus (Supplementary Fig. S3B). This indicated that the majority of the upregulated genes in memory cells responded to either of the stimuli (i.e. TCR or CD28 were capable of driving the response) or they were truly CD28 costimulation dependent, requiring both TCR and CD28 together.

To determine whether genes sensitive to CD28 or TCR regulated the same cellular processes in naive and memory T cells, we tested whether these genes were enriched in hallmark functional pathways [27] (see ‘Materials and methods’ section; Fig. 3C). We observed that DNA replication, as shown by *G2M checkpoint*, was CD28-sensitive in memory cells but TCR-sensitive in naive cells. This was consistent with the differences in proliferation between naive and memory T cells induced by triggering CD28 and TCR (Fig. 1). We observed three genes (*CDC6*, *CDC20* and *CHEK1*) driving the enrichment of the G2M pathway, which were TCR-sensitive in naive cells but switched to CD28 sensitivity in memory cells. We, therefore, sought to identify other ‘switcher’ genes in our dataset, i.e. genes sensitive to a different stimulus between the two cell types. We identified a group of 18 genes that were TCR-sensitive in naive cells and changed to CD28 sensitivity in memory cells. Among these, we identified transferrin receptor *TFRC* (CD71), which is necessary for iron uptake and fuelling of the proliferation, as well as *MCM10* that is an important factor initiating DNA helicase activity and replication (Fig. 3D and Supplementary Table S4). Together these data highlight the enrichment of cell cycle/DNA replication pathways as targets for CD28 in memory T cells which, in contrast, are controlled by the TCR in naive cells. Additionally, these results replicate at the transcriptional level our earlier functional observations that naive and memory T cells display different proliferative responses to TCR and CD28 stimulation.

### T-cell effector functions are predominantly controlled by CD28

**Fig. 4:**
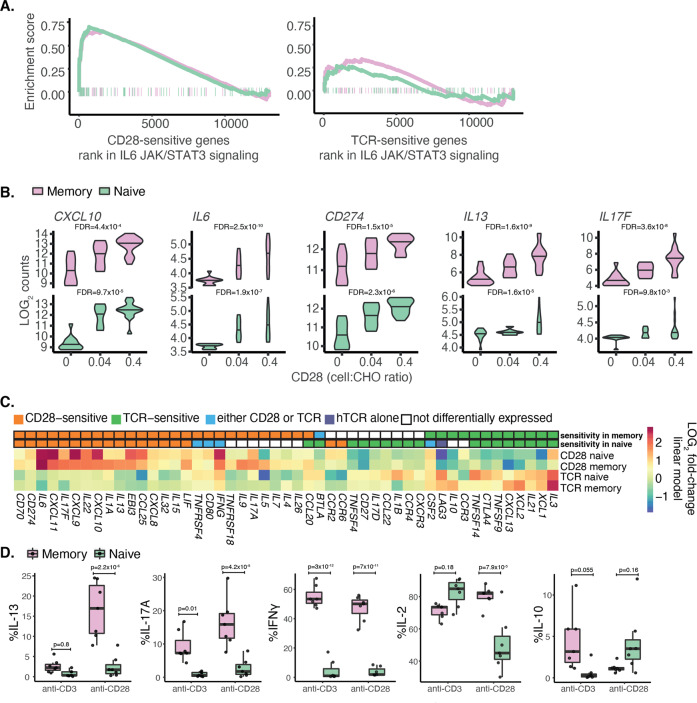
Effector functions of T cells are predominantly controlled by CD28. **A** Pathway enrichment plot for IL6 via JAK/STAT3 response. **B** Examples of cytokines, and chemokines and co-stimulators that are CD28 sensitive in both cell types. The *x* axis corresponds to the level of CD28 (proportion of T cells to CHO-CD86 cells) and the *y* axis corresponds to the log2 counts of gene expression. **C** Cytokines, chemokines and costimulatory molecules that are sensitive to at least one stimulus in at least one cell type. Two upper rows represent stimulus sensitivity in memory or naive T cells. Colouring in the heatmap below represents the log2 fold-change of gene expression based on the linear model per cell type. **D** Purified CTV stained CD4+CD25− memory and naive cells were stimulated by crosslinking anti-CD28 or anti-CD3 antibodies with CHO-FcR cells. The proliferation of memory and naive T cells was measured by flow cytometry five days following stimulation. Shown are the percentage of dividing cells that express IL13, IL17A, IFNɣ, IL2 and IL10. Significance was calculated using two-way ANOVA and group means were compared using Tukey’s honest significant difference test.

We observed that the expression of the majority of genes broadly classified as immune cell pathways, such as *IL6 signalling through the Jak/Stat3*, *inflammatory response* and *interferon α and γ response* were more enriched in CD28-sensitive genes compared to TCR-sensitive genes in both cell types (Fig. 4A). We examined which genes were driving this enrichment and discovered that many cytokines and chemokines were under CD28 control (Fig. 4B). For example, *CXCL10* and *CXCL11* drove the enrichment of all four of these pathways and *IL6* and *IL15RA* drove the enrichment of *IL6 signalling through the Jak/Stat3*, *inflammatory response* and *interferon γ response*. We also identified genes involved in costimulation and cell activation driving the enrichment, such as *CD274* (PD-L1) (*PD1* was not detected in either cell type) driving the enrichment of *interferon ɣ response* and *CD70* driving the enrichment of *inflammatory response*. Since the majority of T-cell stimulation experiments use both TCR and CD28 to activate cells, it is invariably unclear which T-cell functions are controlled by TCR and which by CD28, and how they differ between naive and memory cells. We, therefore, examined all cytokines and chemokines that were expressed in the dataset, as well as selected costimulatory molecules (Fig. 4C). Notably, the expression of cytokines essential for the differentiation of the major Th subsets was found to be under CD28 control in our model, including *IFNG* (Th1), *IL4* and *IL13* (Th2) and *IL17A*, *IL17F* and *IL22* (Th17). In addition, we observed that expression of the Treg transcription factor, *FOXP3*, was also sensitive to CD28 stimulation.

To confirm whether this stimulus selectivity was also present at the protein level, we assessed cytokine expression using flow cytometry (Fig. 4D and Supplementary Fig. S4). We observed that IL-13 (Th2 cytokine) was specifically expressed only in memory T cells in response to CD28 stimulation. On the other hand, IFNɣ (Th1 cytokine) and IL-17A (Th17 cytokine), were expressed only by memory cells and stimulated by either stimulus. However, the percentage of memory T cells expressing IL-17A was higher upon CD28 stimulation than TCR stimulation (*p* = 0.03). Furthermore, CD28 stimulation induced significantly higher IL-2 expression in memory T cells (*p*-value = 7.9 × 10^-5^), consistent with their higher proliferation following CD28 crosslinking (Fig. 1C). Finally, we also observed an unusual trend where IL-10 expression was induced by TCR in memory T cells and increased levels with CD28 in naive T cells (although not statistically significant).

Together, these results indicate that a number of genes associated with effector functions of CD4 T cells are predominantly controlled by the CD28 pathway.

### CD28 and TCR induce changes in T-cell chromatin activity

To gain a better understanding of the gene expression regulation underlying the responses upon TCR and CD28 stimulation, we profiled chromatin accessibility with ATAC-seq [28] and active enhancers and promoters marked by H3K27ac [29]. For this, we compared resting cells with cells stimulated with highTCR alone, highCD28 alone or highTCR and highCD28. Globally, we observed that memory cells were characterised by more peaks in both chromatin accessibility sites (8.6% more peaks; *p*-value = 0.094) and H3K27ac (9.4% more peaks; *p*-value = 0.0016) (Supplementary Fig. 5A).

To understand whether the TCR and CD28 stimuli initiated specific gene expression regulatory programmes, we tested for enrichment of transcription factor binding sites (TFBSs). We used all peaks called per cell type and compared stimulation against the resting state. This revealed a high correlation of enriched TF motifs in chromatin accessible sites between cells stimulated with both TCR and CD28 together and the individual stimuli (Pearson *R*^2^ = 0.86 for CD28 alone and Pearson *R*^2^ = 0.91 for hTCR alone using ATAC-seq). Among the enriched motifs, we identified components of the AP1 transcription factor complex (Supplementary Fig. 5B), which have an important role in the induction of the immune response. Transcription factor motifs for the nuclear receptors family proteins were exclusively enriched in naive T cells stimulated with strong TCR alone, consistent with the induction of anergy in these cells. Similar results were observed using H3K27ac ChM-seq data (Supplementary Fig. 5B).

**Fig. 5:**
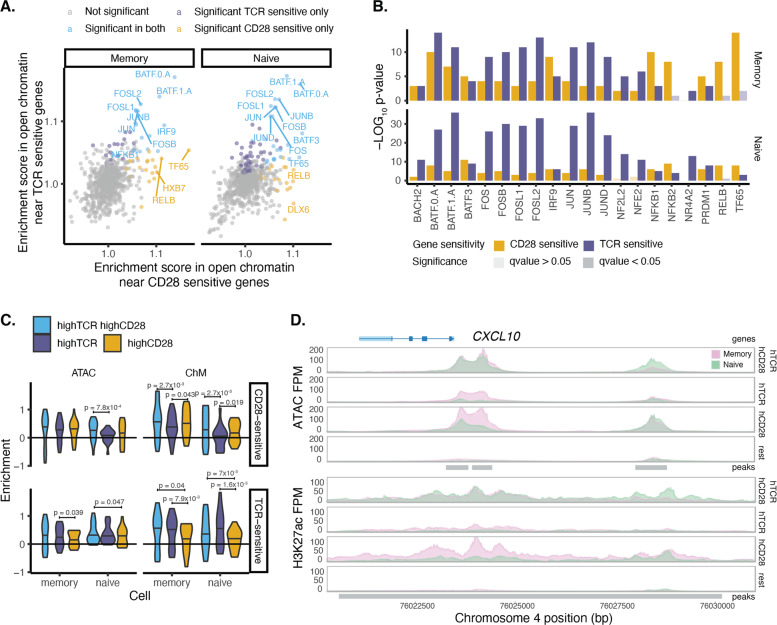
TCR and CD28 signals induce specific changes in chromatin activity. **A** Enrichment score in open chromatin tested using only the open chromatin around stimulus-sensitive genes (taking a 150kb window) and the open chromatin around non-differentially expressed genes in the resting state as background. **B** Top 20 most significantly enriched transcription factors when testing stimulus-sensitive genes. **C** Enrichment in coverage compared to the resting state in the open chromatin around cytokines, chemokines a co-stimulators. Enrichment was calculated as log2(coverage in stimulated cells/coverage in resting cells). *p*-values were calculated using a paired *t*-test. Shown are only *p*-value < 0.05. **D** Selected example of the chromatin around *CXCL10* gene.

We then tested the enriched transcription factor motifs in open chromatin in the proximity of genes that we defined as sensitive to CD28 or TCR. We observed that AP1 transcription factors motifs were enriched in both TCR and CD28-sensitive genes, consistent with the importance of AP1 as a global regulator of T-cell activation (Fig. 5A, B). The enrichment was more significant in TCR-sensitive genes than CD28-sensitive genes, especially in naive cells. In contrast, NF-kB and RELB transcription factors were most significantly enriched in open chromatin near CD28-sensitive genes highlighting NF-kB as a potential mechanism for divergence between TCR and CD28-sensitive genes in line with previous reports [30, 31].

We also examined chromatin accessibility near cytokines and costimulatory genes that we assigned as sensitive to TCR or CD28 signals (Fig. 5C). Chromatin was more open, and the histone acetylation was higher near genes sensitive to CD28 in cells stimulated with a high level of CD28, and similarly near genes sensitive to TCR in cells stimulated with a high level of TCR. We observed a consistent opening of the chromatin upon stimulation around the tested set of genes for 72% of the cases. We could further identify a set of genes that showed differences, consistent with differential sensitivity to TCR and CD28 as well as a high enrichment score (Supplementary Fig. 5C). For example, the chromatin around the TSS of *CXCL10* gene (Fig. 5D) only changed in the presence of CD28 either alone or as a result of costimulation (TCR and CD28 together), whereas TCR alone did not have an effect. On the other hand, the chromatin around *CD28* gene itself changed more in response to highTCR alone in naive T cells, compared to the other two stimuli (Supplementary Fig. 5D). Therefore, for selected genes, the differential effect of TCR or CD28 stimulation on gene expression appears mediated by specific changes in the chromatin organisation and transcription factor recruitment.

### Immune GWAS loci are enriched for CD28-sensitive genes

The role of T-cell activation in the development of immune-mediated diseases is well established and SNPs nearby genes relevant to T-cell activation, differentiation and trafficking have been implicated in disease risk through GWAS [5, 32–34]. We sought to investigate whether immune disease-associated loci were enriched for the genes, we identified as TCR or CD28-sensitive, thereby implicating the involvement of either of these stimulatory pathways in disease pathogenesis.

**Fig. 6:**
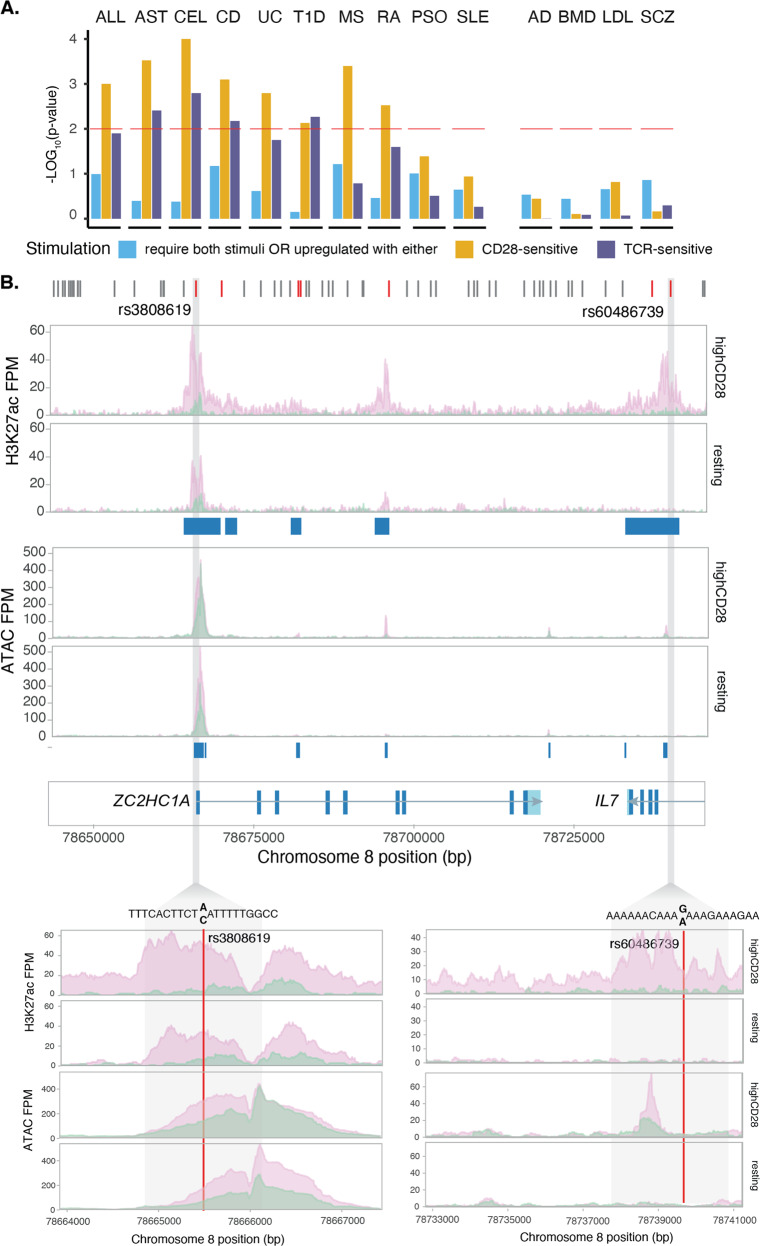
Immune GWAS loci are enriched for CD28-sensitive genes. **A** Enrichment of TCR- and CD28-sensitive genes in immune-mediated disease loci (ALL allergies, AST asthma, CD Crohn’s disease, UC ulcerative colitis, CeD coeliac disease, T1D type-1 diabetes, RA rheumatoid arthritis, SLE systemic lupus erythematosus, MS multiple sclerosis, PSO psoriasis). Alzheimer’s disease (AD), bone mineral density (BMD), LDL cholesterol (LDL) and schizophrenia (SCZ) are used as negative controls. **B** MS-associated locus containing two genes, *ZC2HC1A* and *IL7*, that are CD28-sensitive. In the upper panel are indicated all the SNPs in LD (red) with reported GWAS index variant, rs1021156. Of these, two variants highlighted in grey, rs3808619 and rs60486739, overlap CD28-upregulated H3K27ac peaks and are predicted to disrupt an IRF-binding site.

In our enrichment analysis, we tested ten immune-mediated conditions, allergies (ALL) [35], asthma (AST) [36], coeliac disease (CEL) [37], Crohn’s disease (CD) [37, 38], ulcerative colitis (UC) [38], type-1 diabetes (T1D) [6], multiple sclerosis (MS) [39], rheumatoid arthritis (RA) [5], psoriasis (PSO) [40] and systemic lupus erythematosus (SLE) [41]. We used Alzheimer’s disease (AD) [40, 42], bone mineral density (BMD) [43], LDL cholesterol (LDL) [44] and schizophrenia (SCZ) [45] as negative controls, as we would not expect to observe significant enrichment among loci associated to these traits. The majority of the tested immune diseases showed more significant enrichment (permuted *p*-value < 0.01) for CD28-sensitive genes compared to TCR-sensitive genes. The exception was T1D where genes sensitive to CD28 and TCR both showed a comparable enrichment (Fig. 6A).

The majority of immune disease-associated genetic variants fall in the non-coding regions of the genome and previous studies showed that disease-associated variants are enriched in active enhancers [9, 46]. We, therefore, investigated whether disease SNPs map within active or open chromatin regions as defined by H3K27ac or ATAC peaks near the CD28-sensitive genes driving the enrichment. We observed that, on average across traits, 67% of the genes driving the enrichment also had at least one disease-associated variant overlapping an active promoter or enhancer or chromatin accessible site (Supplementary Fig. 6). We, therefore, tested whether any of these SNPs disrupted a TFBS, limiting our analysis to the TFs that we had previously identified as enriched. We found that 36% of the disease-associated variants overlapping active promoters or enhancers, or open chromatin regions also disrupted a TFBS. The most commonly disrupted motif was the IRF family of transcription factors in regions associated with allergies, Crohn’s disease and multiple sclerosis. Two SNPs associated with MS [39] and in high LD (*r*^2^ > 0.8) with the reported index variant rs1021156 disrupted the IRF TFBS within the *ZC2HC1A*/*IL7* locus (Fig. 6B). One of the variants, rs3808619, is localised in the promoter of *ZC2HC1A* and the risk allele led to decreased binding by IRF family of transcription factors and to increased binding of STAT1 (Fig. 6B). The second variant, rs60486739, is located in intron 3 of *IL7*, and the minor allele led to increased binding by IRF transcription factors (Fig. 6B). Both genes were sensitive to CD28 stimulation in memory cells and the H3K27ac peak that contained the rs60486739 variant was only present in stimulated memory cells, suggesting a potential functional role of the variant in modulating TF binding in this enhancer and affecting the expression levels of the gene.

Together, these results indicate that the variants associated with immune-mediated diseases may affect chromatin activity and by modulating the expression of CD28-sensitive genes steer the outcome of T-cell activation.

## Discussion

The process of T-cell activation is fundamental to the development of immune diseases and a detailed understanding of its control is essential to the design of more effective therapies. Productive T-cell activation involves recognition of antigen by the TCR in association with costimulatory signals via receptors such as CD28 [11]. However, the relative intensities of both signals are likely to be highly variable depending on the setting of T-cell activation. Understanding the requirements for CD28 costimulation during immune responses is important for many therapeutic approaches including immune suppression in autoimmunity and transplantation, as well as cancer immunotherapy.

There is an ongoing debate as to how naive and memory cells differ in their requirements for CD28 costimulation, and a prevalent view is that CD28 costimulation is less required for activation of memory than naive T cells [47–50]. More recently, studies have begun to question this conclusion and have shown CD28 impacts on memory T cells responses in mice [51, 52]. Our study provides additional support for a significant role of CD28 in memory T-cell activation including impacts on human memory CD4^+^ T-cell proliferation and effector functions. We reached this conclusion (i) by using a range of different cellular models where we could manipulate the levels of TCR and CD28, (ii) isolating and profiling only T cells that were actually stimulated therefore reducing the confounding effect of variable activation (iii) separately assessing naive and memory cells as defined by CD45RA expression, and (iv) taking a genome-wide perspective of gene expression through mapping RNA and chromatin changes induced by strong CD28 stimulation. This allowed us to separate the two independent parts of costimulation and identify genes that are expressed in a more TCR or CD28-sensitive manner. By using these multiple approaches our data suggest that whilst both naive and memory T cells use CD28 costimulation to proliferate, memory T cells may in fact be more CD28-sensitive: utilising CD28 to enhance cell cycle, drive chromatin rearrangements affecting specific transcription factors and target T-cell effector cytokines.

It is important to recognise that there are likely to be caveats associated with the models, we have used. In particular, the use of crosslinked CD3 and crosslinked CD28 using antibodies on FcR + CHO cells are unlikely to recapitulate the same cellular responses as these receptors triggered by their natural ligands. Indeed CD3 has no natural ligand and CD28 has two of varying affinity. Nonetheless, antibody ligation has been used to effectively elucidate signals downstream of both TCR and CD28 suggesting the signals themselves can be representative, albeit delivered by unusually high intensity of stimuli that do not occur in physiology. Here we have used CD28 antibody to help delineate CD28 signalling and also to make comparisons with less aggressive stimuli including titrating TCR and CD28 signals via CD86.

Our data indicate that in some settings effective stimulation of memory cells by CD28 alone can occur and whilst unfamiliar they are consistent with several previous observations [23, 53] on CD28 function and suggest this approach may be a legitimate measure of CD28-driven effects. Firstly, the ill-fated CD28 superagonist antibody (TGN1412) trial, which tested the ability of CD28 costimulation to specifically expand and activate Tregs, revealed a powerful response to CD28-driven specifically by effector memory T cells [23]. Secondly, recent data indicated that human Treg cells can also be expanded by utilising CD28 antibodies alone [53]. This is in line with our observations, given that Tregs predominantly consist of memory cells and that CD28 stimulation upregulates *FOXP3*. Thirdly, there is now increasing evidence using conditional deletion of CD28 in mice that memory T-cell responses are dependent on CD28 stimulation [54–57]. Whilst we do not necessarily envisage strong CD28 signals in isolation as a frequent occurrence in biology, our approach serves to identify the downstream impacts of CD28 signalling and not dissimilar to the approach frequently used for anti-CD3 as a proxy for TCR signalling. Indeed, we also used FcR crosslinked anti-CD3 for comparison, observing that a strong TCR signal alone was sufficient to induce the expression of key drivers of cell division and consequently trigger the proliferation of naive T cells as expected. Surprisingly anti-CD3 had a smaller effect on the proliferation of memory T cells. As such, we conclude that memory cells are not simply more sensitive generally to activation signals, but that there is a difference in the TCR and CD28 usage between the two cell types. Our data, therefore, indicate that there is a shift in balance away from TCR and towards CD28 use as cells transit from CD45RA^+^ to CD45RA^−^ status.

The concept that CD28 is involved in the proliferation of memory T cells is intriguing in the light of recent data related to checkpoint blockade for cancer treatment. It has been suggested that PD-1 blockade, which is important to the reinvigoration of exhausted effector T cells, requires CD28 signalling [58, 59]. Again, this aligns well with our data and supports the concept that differentiated memory T cells in tumours utilise CD28. Our findings are also consistent with the fact that CTLA-4 and PD1 blockade, both of which increase CD28 signals, are known to trigger autoimmunity [60, 61].

Finally, our findings have further implications for understanding susceptibility to complex immune-mediated diseases, where T-cell activation is one of the hallmark pathobiological processes. GWAS of immune diseases has mapped hundreds of associated risk loci, many of which harbour genes of immune function. However, the specific role of the identified genes in T-cell activation processes is unclear. By examining T-cell gene expression sensitivity in response to specific stimuli, we demonstrated that GWAS loci are enriched for CD28-sensitive genes, rather than TCR-sensitive genes, thereby increasing support for the role of T-cell activation via CD28 costimulation in susceptibility of immune-mediated diseases.

For example, a recent study identified that cytokine oncostatin M (OSM) is expressed at higher levels in inflamed intestinal tissues from IBD patients compared to healthy controls [62]. In our dataset, *OSM* is CD28-sensitive and is among the genes driving the enrichment of CD28-sensitive genes in CD and UC. The importance of CD28 costimulation in immune-mediated diseases is further supported by data from the CTLA-4 field [63]. Loss of CTLA-4 in mice and heterozygous mutations in humans reveal profound autoimmunity where enteropathy is a consistent feature [14, 16, 17]. The fact that the CTLA-4 pathway directly regulates CD28 stimulation by competing for the same ligands [13] means that CD28 is a direct driver of these autoimmune phenotypes. Nonetheless, given that CD28 activation affects multiple other costimulatory pathways and effector functions the importance of these downstream targets needs to be considered when interpreting CD28 impacts. Furthermore, it is possible that some of these genes are also sensitive to other costimulatory molecules that were not investigated in this study, highlighting the importance to carry similar experiments with a range of co-stimulators.

Taken together, our study provides new insights into the role of TCR and CD28 costimulation in the activation and proliferation of human naive and memory CD4 T cells, and the influence of these stimuli on immune disease susceptibility.

## Materials and methods

### Sample collection and DNA isolation

Blood samples were obtained from eight healthy adults, aged from 22 to 46 years. Peripheral blood mononuclear cells (PBMCs) were isolated using Ficoll-Paque PLUS (GE healthcare, Buckingham, UK) density gradient centrifugation. CD4^+^ T cells were isolated from PBMCs using the negative selection EasySep® CD4^+^ enrichment kit (StemCell Technologies, Meylan, France) according to the manufacturer’s instructions. DNA was isolated from live PBMCs using Qiagen DNeasy blood and tissue kit.

Samples used for RNA extraction analysis were obtained in accordance with the commercial vendor’s approved institutional review board protocols. The sample used for ATAC-seq and H3K27ac ChM-seq were obtained from NHSBT Cambridgeshire. All research use was approved by the Research Ethics Committee (reference number: 15/NW/0282).

#### Cell line engineering

CRISPR-Cas9 targeting was used for the generation of CD80/86-KO DG-75 lines. sgRNAs were designed using CHOPCHOP (https://chopchop.rc.fas.harvard.edu). sgRNA syntheses containing a target sequence for CD80 (TTGAGGTATGGACACTTGGA) or CD86 (TTGACCTGCTCATCTATACA) were performed using the EnGen sgRNA Synthesis Kit, *S. pyogenes* (NEB) according to the manufacturer’s instructions. sgRNAs were purified using the RNA Clean & Concentrator kit (Zymo Research) following the manufacturer’s instructions. The cell line was generated as follows: 500 ng sgRNA and 2 μg Cas9 protein (TrueCut™ Cas9 Protein v2, ThermoFisher Scientific) were electroporated into 2 × 10^5^ DG-75 cells using the Neon™ Transfection System (Thermo Fisher Scientific) Transfection was carried out using 10-μl tip, and Buffer R as indicated: voltage (1600 V), width (10 ms), pulses (three). Cells were allowed to recover for 3–5 days prior to screening for KO by flow cytometry. This approach generally yielded KO of the target gene in 70–95% of the cells. These cell populations were then sorted based on loss of expression of the target.

Transduced cell lines were generated using the MP71 retroviral vector containing CD86 GFP-tagged fusion proteins. Retroviral supernatants were generated by transfection of Phoenix-Amphoteric packaging cells, using the FUGENE HD transfection reagent (Roche Molecular Biochemical). Twenty-four hours post-transfection, viral supernatants were harvested and used to transduce the DG-75 B cell line. For transduction, non-tissue culture treated 24-well plates were coated with RetroNectin (TaKaRa) for 2 h at RT at 30 mg/ml. 3 × 10^5^ DG-75 cells per well were added to 1 ml of retroviral supernatant and centrifuged at 2000 rpm, 32 °C for 2 h. Twenty-four hours post-infection, viral supernatant was removed and fresh media added. Three days post-transduction, cells were screened by flow cytometry for transduced protein expression.

### Cell culture and stimulation

Prior to stimulation CD4^+^CD25^−^ naive and memory T cells were purified using EasySep Human Naïve CD4^+^ T cell Isolation kit and EasySep Human Memory CD4^+^ T Cell Enrichment kit respectively, followed by CD25 depletion using the custom EasySep Human CD4^+^CD25^−^ Isolation kit. Isolations were performed according to the manufacturer’s instructions (STEMCELL Technologies). Cells were labelled with CellTrace Violet dye (Life Technologies) according to the manufacturer’s instructions. For stimulation with the DG75 B cells, CTV labelled memory and naive T cells were stimulated for 5 days in the presence of CD86 transduced DG75 B cells at a T:B ratio of 1:0.5. TSST-1 superantigen (0.25 μg/ml) or OKT3 (1, 0.01 and 0.0001 μg/ml) was used to stimulate TCR and different concentrations of abatacept (0.2, 2 and 20 μg/ml) to block CD28 costimulation. Proliferation of CD4 stained (CD4 AF700 Clone RPA-T4) Vβ2^+^ T cells (TCR Vβ2-PE, Clone MPD2D5, Beckman Coulter) was determined via flow cytometry and the number of cells within the dividing gate calculated using AccuCheck counting beads (Invitrogen) and FLOWJO proliferation software.

Chinese hamster ovary (CHO) cells expressing CD86 or FcR (FcRγII, CD32) were cultured in DMEM (Life Technologies, Paisley, UK) supplemented with 10% v/v FBS (Sigma, Gillingham, UK), 50 U/ml penicillin and streptomycin (Life Technologies), and 200 μM l-glutamine (Life Technologies) and incubated at 37 °C in a humidified atmosphere of 5% CO2. CHO cells expressing CD86 and FcR were generated as previously described [13]. CD4^+^CD25^−^ T cells were co-cultured with glutaraldehyde fixed CHO-CD86 to provide CD28 signal or CHO-FcR for 16 h in RPMI 1640 supplemented with 10% v/v FBS, 50 U/ml penicillin and streptomycin, and 200 μM l-glutamine. This time point was chosen because T cells upregulate activation markers such as CD25, but they do not proliferate. Following 16 h stimulation, we sorted activated CD25^+^ cells into naive (CD45RA^+^) and memory (CD45RA^-^) subsets on which we performed RNA-seq, ATAC-seq and H3K27ac-seq. Cultures stimulated with CHO-CD86 were treated with anti-CD3 (clone OKT3). Here, high TCR corresponds to 1 ug/ml, low TCR corresponds to 0.01 μg/ml; high CD28 corresponds to 1:2.5 CHO-CD86 to T-cell ratio and low CD28 corresponds to 1:25 CHO-CD86 to T-cell ratio. Cultures stimulated with CHO-FcR were treated with 1 µg/ml of anti-CD3 (OKT3) or 1 µg/ml of anti-CD28 (9.3) (Supplementary Fig. S1B). We have previously shown that on their own CHO cells do not activate T cells [30, 64]. Indeed, in our experimental setup, we used resting cells where T cells were cultured with fixed CHO cells and we observed no cell activation, as shown by the lack of CD25 upregulation (Supplementary Fig. S1C).

### Flow cytometry

CD4^+^ enriched cells were stained with the following antibodies for sorting: CD4 (OKT4)-APC (Biolegend); CD25 (M-A251)-PE (Biolegend); CD127 (eBioRDR5)-FITC (eBioscience) and Live/Dead fixable blue dead cell stain. None of these antibodies have been reported to be agonistic. Live conventional T cells (Tcons, CD4^+^CD25low CD127high) were isolated and cultured for 16 h. Stimulated naive and memory cells were sorted based on the expression of CD25-PE, CD45RA- Alexa700 (Biolegend) and DAPI. Resting naive and memory cells, we sorted for low expression of CD25 (proportion of CD25^+^ cells < 1%, see Supplementary Fig. S1).

For intracellular cytokine staining, following 5 days of culture, cells were re-stimulated with 50 ng/ml phorbol myristate acetate (PMA) (Sigma), 1 μM Ionomycin (Sigma) and 10 μg/ml Brefeldin A (Sigma) for 4 h at 37 °C. Following incubation, cells were stained with Near IR viability dye (ThermoFisher) at a 1 in 2000 dilution in PBS, fixed and permeabilised using the FoxP3 Transcription Factor Staining Buffer Set according to protocol A of manufacturers instructions (ThermoFisher). All stains were performed on ice. Cells were stained with the following antibodies; CD4 PE-CF594 CloneL200, IL13 Bv711 Clone JES10-5A2, IFNγ BUV395 Clone B27, IL17A Bv650 Clone N49-653, IL2 PE-Cy7 Clone MQ1-17H12 and IL10 APC Clone JES3-19F1.

### RNA-seq

Naive and memory T cells were placed in 0.5 ml of Trizol (Invitrogen) and stored at −80 °C. Samples were thawed at 37 °C before adding 100 μl chloroform. After reaching equilibrium, samples were centrifuged for 15 min at 4 °C at 10,000 × *g*. The collected aqueous phase was mixed 1:1 with 70% ethanol before proceeding with minElute columns (Qiagen) for purification, according to the manufacturer’s protocol. RNA was quantified using the Agilent Bioanalyzer. The purified RNA was sequenced in two separate batches. Libraries were prepared using Illumina TruSeq index tags and sequenced on the Illumina HiSeq 2500 platform using V4 chemistry and standard 75 bp paired-end. The first batch consisted of 56 samples that were multiplexed at equimolar concentrations and sequenced across 14 lanes, to yield on average 71.3 million reads per sample. These 56 samples included four donors per condition per cell type (4 donors × 7 conditions × 2 cell types). The second batch consisted of 18 samples that were multiplexed at equimolar concentrations and sequenced across three lanes to yield on average 61 million reads per sample. These samples included four donors stimulated with hCD28 or hTCR in memory cells, three donors stimulated with hCD28 or hTCR in naive cells and two donors in the resting state for both memory and naive cells.

### RNA-seq data processing

Paired sequence reads were aligned to the GRCh38 human reference genome using STAR (v2.5.0c) [65] and the Ensembl reference transcriptome (v83). Gene counts were estimated using featureCounts (v1.5.1) tools [66] from the subread package and only reads assigned to the transcripts were used for further processing (84–90% of reads were assigned). We only kept genes from autosomal chromosomes and chromosome X and genes with at least 20 copies in at least three samples. This left us with a total of 13,246 genes.

We used raw input count data for DESeq2 analysis since DESeq2 uses a built-in negative binomial distribution model. The resulting beta prior variance $\sigma _d^2$ = 0.661 and the dispersion prior variance $\sigma _d^2$ = 0.761. To find genes that were upregulated upon stimulation, we first defined differentially expressed genes by performing a pairwise comparison of all the conditions to the resting state, in a cell type-specific manner, using Benjamini-Hochberg controlled FDR of 5% and an absolute fold-change ≥ 2. We then build a linear and a switch model of gene expression using the LRT algorithm of DESeq2 (v1.14.1) [67] separately for naive and memory cells. In the linear model, we assumed a linear increase of gene expression along with stimulus intensity (incremental fold-change ≥ 1.5 in gene expression). Genes that did not follow the linear model were tested for the switch model. Here, we assumed an “on-and-off” mode of expression, where a gene is significantly upregulated (fold-change ≥ 2) in response to the presence of either CD28 or TCR (Fig. 1B). In both of these models, we used all seven conditions, e.g. when testing for CD28-sensitive genes, we grouped the TCR alone stimulation with the resting since neither received CD28 signal. A gene was classified in one of the two categories without overlap and prioritised for the linear model.

To control for the different batches in which we processed the blood, which accounted for 12% of the observed variability, we performed batch correction prior to PCA using the combat algorithm as implemented by the sva package [68]. To estimate the percentage of the variance explained separately by each of the recorded variables, such as the stimulus, the cell type and the gender of the donors among others, we fitted a linear model with only the stimulus or the cell type as variables (method adapted from ref. [69]).

We performed pathway enrichment analysis using the fgsea package [70] on R Bioconductor. We tested whether different gene-sets were over-represented in GSEA hallmark pathways [27, 68] using the linear model derived fold-change rank. We used 1,000,000 permutations to derive corrected p-values.

### ChIPmentation-seq (ChM-seq)

ChIPmentation-seq was performed according to a published protocol [29], with the following modifications to make it compatible with the iDeal Histone ChIP kit (Diagenode) buffers. Five hundred thousand crosslinked cells were washed using 250 μl IL1 buffer and resuspended in 250 μl IL2 lysis buffer, both of which contained 1× protease inhibitor cocktail (PIC). Cells were left to lyse for 5 min at 4 °C on a rotator and then centrifuged at 4 °C (3000×*g*) for 5 min. The pellets were resuspended in 250 μl IS1 buffer and sonicated using the Bioruptor®Pico (Diagenode, Belgium) for 5 min for resting cells or 4 min for stimulated cells (Diagenode). We kept a portion of the chromatin from two samples aside, one naive and one memory stimulated with highTCR and highCD28, and used them as a ChM-seq input.

The chromatin was immunoprecipitated using protein-A coated IP beads. Twenty microliters of beads were washed four times using 40 μl IC1 buffer on the magnetic rack before being resuspended in 20 μl of IC1. The beads were mixed with 56 μl 5× IC1 buffer, 6 μl 50X BSA, 1.5 μl 200× PIC and 1ug antibody. We added to the mix 100 μl of chromatin (equivalent to 200,000 cells) and incubated the samples overnight at 4 °C at 10 rpm.

The beads were then washed on the magnet with 350 μl of iW1, iW2 and iW3 buffers and a final wash with 2 × 1000 μl 10 mM Tris pH 8. The beads with the chromatin, as well as the two input samples, were then resuspended in 29 μl ChM buffer (Tris pH 8 1 M, MgCl2 1 M, ChIP grade water) with 1 μl Tn5 and incubated for 10 min at 37 °C at 1500 rpm. The tagmentation was stopped with the addition of 2 × 350 μl iW3. Finally, the beads were washed with 350 μl iW4. The chromatin from the beads was eluted using 96 μl iE1 and incubated for 30 min at room temperature at 1500 rpm. The chromatin was reverse crosslinked overnight using 4 μl of iE2 buffer. The DNA was then purified using the MinElute PCR CLeanup kit (QIAGEN) and eluted in 30 μl of water. Sequencing libraries were prepared using Nextera primers as described in the ATAC-seq protocol [28]. Eighteen libraries were indexed and pooled in equimolar concentration and sequenced on three lanes using the Illumina HiSeq 2500 platform and V4 chemistry using standard 75 bp paired-end reads to yield on average 80 million reads per sample.

### ATAC-seq

ATAC-seq was performed according to a published protocol [28], with a modification to reduce the number of mitochondrial reads. Fifty thousand cells were washed with 1 ml of ice-cold phosphate-buffered saline (PBS). The cells were then resuspended in the tagmentation buffer containing Tn5 transposase (Nextera, Illumina) and 0.01% digitonin and incubated for 30 min at 37 °C before purifying the DNA using the MinElute PCR purification kit (QIAGEN). Sequencing libraries were prepared using Nextera primers as described in the ATAC-seq protocol [28]. Sixteen libraries were indexed and pooled in equimolar concentration and sequenced on three lanes using the Illumina HiSeq 2500 platform and V4 chemistry using standard 75 bp paired-end reads to yield on average 65 million reads per sample.

### ChM and ATAC data processing

The quality of the sequence reads was assessed using the fastx toolkit and the adaptors were trimmed using skewer (v0.2.2) [71]. Reads were mapped to the human genome reference GRCh38 using the bwa mem algorithm (v0.7.9a) [72]. We only kept uniquely mapped reads, removed PCR duplicated reads and for the ATAC, we excluded mitochondrial reads using samtools (v0.1.9) [72, 73]. We retained 83.3% of ATAC and 73.8% of ChM reads. Genome browser tracks were created using BEDTools (v2.22.0) [74] and the UCSC binary utilities. Furthermore, we generated insert size distributions using PICARD tools (v2.6.0) CollectInsertSizeMetrics function which can be indicative of over-sonicated chromatin and excess of adapters in the data. The mapped reads were converted into bed files and chimeras were removed. Peaks were called using MACS2 (v2.1.1) [75] setting the parameters to -q 0.05 -nomodel -extsize 200 -shift −100 for ATAC, and -broad -broad-cutoff 0.1 -nomodel -extsize 146 for H3K27ac ChM. For ChM, all samples were downsampled to the same read number (21.6 million reads) prior to peak calling against the input.

We used the fraction of reads in peaks (FRiP), the proportion of peaks with signal value (fold-change compared to the background or the input) greater than 10, the insert size distribution and the genome tracks to investigate the quality of our data. The median FRiP score for ATAC was 59.2% and 73.7% for H3K27ac ChM. The proportion of peaks with fold-change >10 was 22.9% for ATAC and 1.7% for ChM.

### Transcription factor motif enrichment

Global transcription factor enrichment was carried using the top 70,000 most significant peaks for ATAC and the top 50,000 peaks for ChM-seq per cell type-stimulation. As a background, the resting state of each cell type was used. We used the *findMotifsGenome.pl* function from the Homer suite [76], setting the genome reference as hg38, the –size as 200 for ATAC-seq and 1000 for ChM-seq and without computing novel motifs. We used the HOCOMOCOv11 Human database of motifs. Transcription factor enrichment near signal sensitive genes was done by taking a 150 kb window around each gene and intersecting the gene boundaries with the peaks called in each assay using bedtools intersect. As a background, we used the peaks intersecting non-differentially expressed genes.

The enrichment of cytokine reads was done by taking the same 150 kb window around cytokine, chemikine and costimulatory molecules and counting the number of reads falling within these boundaries. The coverage was then calculated by dividing by the total read number. Enrichment was calculated by taking the logarithm of the stimulated state for a gene over the resting state.

### Disease SNP enrichment for stimulus-sensitive genes

From the GWAS data, we excluded all variants that fell within the MHC locus and used a genome-wide *p*-value threshold of <5 × 10^−8^. We defined the disease loci by mapping all the SNPs in linkage disequilibrium (LD) with the reported index SNP, using *r*^2^ > 0.8 calculated across the European populations present in the 1000 Genomes Project data, and extending the LD boundaries by 150 kb on each side, to account for the possibility of distant gene expression regulation between enhancers and gene promoters. This resulted in 334 unique regions associated to one of the ten tested traits.

We then tested whether the stimulus-sensitive genes that were defined in the linear and the switch models fell within the SNP loci boundaries more often than expected by chance using a permutation strategy. To build our null distribution, we selected the same number of genes, matching for gene size and expression level. We repeated the process 10,000 times.

We tested whether any of the SNPs used to define the LD boundaries overlapped with an H3K27ac or an ATAC peak identified in any of the conditions. The disruption of TFBS by SNPs was assessed using the SNP2TFBS database [77, 78].

## Supplementary information

Supplementary Tables and Figures legends

Suppl. Figure 1

Suppl. Figure 2

Suppl. Figure 3

Suppl. Figure 4

Suppl. Figure 5

Suppl. Figure 6

Suppl. Table 1

Suppl. Table 2

Suppl. Table 3

Suppl. Table 4

## Data Availability

The accession numbers for the sequencing data reported in this paper are EGAS00001002438 (RNA-seq), EGAS00001002599 (H3K27ac ChM-seq) and EGAS00001004147 (ATAC-seq) and can be accessed through the European Genome-Phenome Archive (EGA; http://ega-archive.org). All the codes used in the data analysis are available through github: https://github.com/trynkaLab/T-cell-costimulation and the results can be browsed at www.sanger.ac.uk/science/tools/costimulation/costimulation.

## References

[1] Haufe S, Haug M, Schepp C, Kuemmerle-Deschner J, Hansmann S, Rieber N (2011). Impaired suppression of synovial fluid CD4+CD25- T cells from patients with juvenile idiopathic arthritis by CD4+CD25+ Treg cells. Arthritis Rheum.

[2] Kshirsagar S, Binder E, Riedl M, Wechselberger G, Steichen E, Edelbauer M (2013). Enhanced activity of Akt in Teff cells from children with lupus nephritis is associated with reduced induction of tumor necrosis factor receptor-associated factor 6 and increased OX40 expression. Arthritis Rheum.

[3] Fortune MD, Guo H, Burren O, Schofield E, Walker NM, Ban M (2015). Statistical colocalization of genetic risk variants for related autoimmune diseases in the context of common controls. Nat Genet.

[4] Dubois PCA, Trynka G, Franke L, Hunt KA, Romanos J, Curtotti A (2010). Multiple common variants for celiac disease influencing immune gene expression. Nat Genet.

[5] Okada Y, Wu D, Trynka G, Raj T, Terao C, Ikari K (2014). Genetics of rheumatoid arthritis contributes to biology and drug discovery. Nature.

[6] Onengut-Gumuscu S, Chen W-M, Burren O, Cooper NJ, Quinlan AR, Mychaleckyj JC (2015). Fine mapping of type 1 diabetes susceptibility loci and evidence for colocalization of causal variants with lymphoid gene enhancers. Nat Genet.

[7] Fairfax BP, Humburg P, Makino S, Naranbhai V, Wong D, Lau E (2014). Innate immune activity conditions the effect of regulatory variants upon monocyte gene expression. Science.

[8] Westra H-J, Peters MJ, Esko T, Yaghootkar H, Schurmann C, Kettunen J (2013). Systematic identification of trans eQTLs as putative drivers of known disease associations. Nat Genet.

[9] Calderon D, Nguyen MLT, Mezger A, Kathiria A, Müller F, Nguyen V, et al. Landscape of stimulation-responsive chromatin across diverse human immune cells. Nat Genet. 2019. 10.1038/s41588-019-0505-9. 10.1038/s41588-019-0505-9 PMC6858557 31570894

[10] Soskic B, Cano-Gamez E, Smyth DJ, Rowan WC, Nakic N, Esparza-Gordillo J (2019). Chromatin activity at GWAS loci identifies T cell states driving complex immune diseases. Nat Genet.

[11] Esensten JH, Helou YA, Chopra G, Weiss A, Bluestone JA (2016). CD28 costimulation: from mechanism to therapy. Immunity.

[12] Rowshanravan B, Halliday N, Sansom DM (2018). CTLA-4: a moving target in immunotherapy. Blood.

[13] Qureshi OS, Zheng Y, Nakamura K, Attridge K, Manzotti C, Schmidt EM (2011). Trans-endocytosis of CD80 and CD86: a molecular basis for the cell-extrinsic function of CTLA-4. Science.

[14] Tivol EA, Borriello F, Schweitzer AN, Lynch WP, Bluestone JA, Sharpe AH (1995). Loss of CTLA-4 leads to massive lymphoproliferation and fatal multiorgan tissue destruction, revealing a critical negative regulatory role of CTLA-4. Immunity.

[15] Lo B, Zhang K, Lu W, Zheng L, Zhang Q, Kanellopoulou C (2015). AUTOIMMUNE DISEASE. Patients with LRBA deficiency show CTLA4 loss and immune dysregulation responsive to abatacept therapy. Science.

[16] Schubert D, Bode C, Kenefeck R, Hou TZ, Wing JB, Kennedy A (2014). Autosomal dominant immune dysregulation syndrome in humans with CTLA4 mutations. Nat Med.

[17] Kuehn HS, Ouyang W, Lo B, Deenick EK, Niemela JE, Avery DT (2014). Immune dysregulation in human subjects with heterozygous germline mutations in CTLA4. Science.

[18] Tivol EA, Boyd SD, McKeon S, Borriello F, Nickerson P, Strom TB (1997). CTLA4Ig prevents lymphoproliferation and fatal multiorgan tissue destruction in CTLA-4-deficient mice. J Immunol.

[19] Tai X, Van Laethem F, Sharpe AH, Singer A (2007). Induction of autoimmune disease in CTLA-4-/- mice depends on a specific CD28 motif that is required for in vivo costimulation. Proc Natl Acad Sci USA.

[20] Gardner D, Jeffery LE, Sansom DM (2014). Understanding the CD28/CTLA-4 (CD152) pathway and its implications for costimulatory blockade. Am J Transpl.

[21] Borriello F, Sethna MP, Boyd SD, Schweitzer AN, Tivol EA, Jacoby D (1997). B7-1 and B7-2 have overlapping, critical roles in immunoglobulin class switching and germinal center formation. Immunity.

[22] Eastwood D, Findlay L, Poole S, Bird C, Wadhwa M, Moore M (2010). Monoclonal antibody TGN1412 trial failure explained by species differences in CD28 expression on CD4+ effector memory T-cells. Br J Pharm.

[23] Hünig T (2012). The storm has cleared: lessons from the CD28 superagonist TGN1412 trial. Nat Rev Immunol.

[24] Weng N-P, Araki Y, Subedi K (2012). The molecular basis of the memory T cell response: differential gene expression and its epigenetic regulation. Nat Rev Immunol.

[25] Kimmig S, Przybylski GK, Schmidt CA, Laurisch K, Möwes B, Radbruch A (2002). Two subsets of naive T helper cells with distinct T cell receptor excision circle content in human adult peripheral blood. J Exp Med.

[26] Fraser J, Irving B, Crabtree G, Weiss A (1991). Regulation of interleukin-2 gene enhancer activity by the T cell accessory molecule CD28. Science.

[27] Liberzon A, Birger C, Thorvaldsdóttir H, Ghandi M, Mesirov JP, Tamayo P (2015). The Molecular Signatures Database (MSigDB) hallmark gene set collection. Cell Syst.

[28] Buenrostro JD, Giresi PG, Zaba LC, Chang HY, Greenleaf WJ (2013). Transposition of native chromatin for fast and sensitive epigenomic profiling of open chromatin, DNA-binding proteins and nucleosome position. Nat Methods.

[29] Schmidl C, Rendeiro AF, Sheffield NC, Bock C (2015). ChIPmentation: fast, robust, low-input ChIP-seq for histones and transcription factors. Nat Methods.

[30] Edmead CE, Patel YI, Wilson A, Boulougouris G, Hall ND, Ward SG (1996). Induction of activator protein (AP)-1 and nuclear factor-kappaB by CD28 stimulation involves both phosphatidylinositol 3-kinase and acidic sphingomyelinase signals. J Immunol.

[31] Takeda K, Harada Y, Watanabe R, Inutake Y, Ogawa S, Onuki K (2008). CD28 stimulation triggers NF-κB activation through the CARMA1–PKCθ–Grb2/Gads axis. Int Immunol.

[32] Trynka G, Sandor C, Han B, Xu H, Stranger BE, Liu XS (2013). Chromatin marks identify critical cell types for fine mapping complex trait variants. Nat Genet.

[33] Farh KK-H, Marson A, Zhu J, Kleinewietfeld M, Housley WJ, Beik S (2015). Genetic and epigenetic fine mapping of causal autoimmune disease variants. Nature.

[34] Hu X, Kim H, Raj T, Brennan PJ, Trynka G, Teslovich N (2014). Regulation of gene expression in autoimmune disease loci and the genetic basis of proliferation in CD4+ effector memory T cells. PLoS Genet.

[35] Ferreira MA, Vonk JM, Baurecht H, Marenholz I, Tian C, Hoffman JD (2017). Shared genetic origin of asthma, hay fever and eczema elucidates allergic disease biology. Nat Genet.

[36] Demenais F, Margaritte-Jeannin P, Barnes KC, Cookson WOC, Altmüller J, Ang W (2018). Multiancestry association study identifies new asthma risk loci that colocalize with immune-cell enhancer marks. Nat Genet.

[37] Trynka G, Hunt KA, Bockett NA, Romanos J, Mistry V, Szperl A (2011). Dense genotyping identifies and localizes multiple common and rare variant association signals in celiac disease. Nat Genet.

[38] Jostins L, Ripke S, Weersma RK, Duerr RH, McGovern DP, Hui KY (2012). Host-microbe interactions have shaped the genetic architecture of inflammatory bowel disease. Nature.

[39] Beecham AH, Patsopoulos NA, Xifara DK, Davis MF, Kemppinen A (2013). Analysis of immune-related loci identifies 48 new susceptibility variants for multiple sclerosis. Nat Genet.

[40] Tsoi LC, Stuart PE, Tian C, Gudjonsson JE, Das S, Zawistowski M (2017). Large scale meta-analysis characterizes genetic architecture for common psoriasis associated variants. Nat Commun.

[41] Langefeld CD, Ainsworth HC, Cunninghame Graham DS, Kelly JA, Comeau ME, Marion MC (2017). Transancestral mapping and genetic load in systemic lupus erythematosus. Nat Commun.

[42] Jansen IE, Savage JE, Watanabe K, Bryois J, Williams DM, Steinberg S (2019). Genome-wide meta-analysis identifies new loci and functional pathways influencing Alzheimer’s disease risk. Nat Genet.

[43] Kemp JP, Morris JA, Medina-Gomez C, Forgetta V, Warrington NM, Youlten SE (2017). Identification of 153 new loci associated with heel bone mineral density and functional involvement of GPC6 in osteoporosis. Nat Genet.

[44] Hoffmann TJ, Theusch E, Haldar T, Ranatunga DK, Jorgenson E, Medina MW (2018). A large electronic-health-record-based genome-wide study of serum lipids. Nat Genet.

[45] Schizophrenia Working Group of the Psychiatric Genomics Consortium. (2014). Biological insights from 108 schizophrenia-associated genetic loci. Nature.

[46] Soskic B, Cano-Gamez E, Smyth DJ, Rowan WC, Nakic N, Esparza-Gordillo J (2019). Chromatin activity at GWAS loci identifies T cell states driving complex immune diseases. Nat Genet.

[47] Dubey C, Croft M, Swain SL (1995). Costimulatory requirements of naive CD4+ T cells. ICAM-1 or B7-1 can costimulate naive CD4 T cell activation but both are required for optimum response. J Immunol.

[48] London CA, Lodge MP, Abbas AK (2000). Functional responses and costimulator dependence of memory CD4+ T cells. J Immunol.

[49] Croft M, Bradley LM, Swain SL (1994). Naive versus memory CD4 T cell response to antigen. Memory cells are less dependent on accessory cell costimulation and can respond to many antigen-presenting cell types including resting B cells. J Immunol.

[50] Luqman M, Bottomly K (1992). Activation requirements for CD4+ T cells differing in CD45R expression. J Immunol.

[51] Borowski AB, Boesteanu AC, Mueller YM, Carafides C, Topham DJ, Altman JD (2007). Memory CD8+ T cells require CD28 costimulation. J Immunol.

[52] Fuse S, Zhang W, Usherwood EJ (2008). Control of memory CD8+ T cell differentiation by CD80/CD86-CD28 costimulation and restoration by IL-2 during the recall response. J Immunol.

[53] He X, Smeets RL, van Rijssen E, Boots AMH, Joosten I, Koenen HJPM (2017). Single CD28 stimulation induces stable and polyclonal expansion of human regulatory T cells. Sci Rep.

[54] van der Heide V, Homann D (2016). CD28 days later: resurrecting costimulation for CD8(+) memory T cells. Eur J Immunol.

[55] Linterman MA, Denton AE, Divekar DP, Zvetkova I, Kane L, Ferreira C, et al. CD28 expression is required after T cell priming for helper T cell responses and protective immunity to infection. Elife. 2014;3. 10.7554/elife.03180. 10.7554/eLife.03180 PMC4241536 25347065

[56] Ndlovu H, Darby M, Froelich M, Horsnell W, Lühder F, Hünig T (2014). Inducible deletion of CD28 prior to secondary *Nippostrongylus brasiliensis* infection impairs worm expulsion and recall of protective memory CD4 T cell responses. PLoS Pathog.

[57] Fröhlich M, Gogishvili T, Langenhorst D, Lühder F, Hünig T (2016). Interrupting CD28 costimulation before antigen rechallenge affects CD8(+) T-cell expansion and effector functions during secondary response in mice. Eur J Immunol.

[58] Hui E, Cheung J, Zhu J, Su X, Taylor MJ, Wallweber HA (2017). T cell costimulatory receptor CD28 is a primary target for PD-1-mediated inhibition. Science.

[59] Kamphorst AO, Wieland A, Nasti T, Yang S, Zhang R, Barber DL (2017). Rescue of exhausted CD8 T cells by PD-1-targeted therapies is CD28-dependent. Science.

[60] Ben Nasr M, Tezza S, D’Addio F, Mameli C, Usuelli V, Maestroni A et al. PD-L1 genetic overexpression or pharmacological restoration in hematopoietic stem and progenitor cells reverses autoimmune diabetes. Sci Transl Med. 2017;9. 10.1126/scitranslmed.aam7543. 10.1126/scitranslmed.aam7543 PMC6171337 29141886

[61] Bertrand A, Kostine M, Barnetche T, Truchetet M-E, Schaeverbeke T (2015). Immune related adverse events associated with anti-CTLA-4 antibodies: systematic review and meta-analysis. BMC Med.

[62] West NR, Hegazy AN, Owens BMJ, Bullers SJ, Linggi B, Buonocore S (2017). Oncostatin M drives intestinal inflammation and predicts response to tumor necrosis factor-neutralizing therapy in patients with inflammatory bowel disease. Nat Med.

[63] Walker LSK, Sansom DM (2015). Confusing signals: recent progress in CTLA-4 biology. Trends Immunol.

[64] Manzotti CN, Liu MKP, Burke F, Dussably L, Zheng Y, Sansom DM (2006). Integration of CD28 and CTLA-4 function results in differential responses of T cells to CD80 and CD86. Eur J Immunol.

[65] Dobin A, Davis CA, Schlesinger F, Drenkow J, Zaleski C, Jha S (2013). STAR: ultrafast universal RNA-seq aligner. Bioinformatics.

[66] Liao Y, Smyth GK, Shi W (2014). featureCounts: an efficient general purpose program for assigning sequence reads to genomic features. Bioinformatics.

[67] Love MI, Huber W, Anders S (2014). Moderated estimation of fold change and dispersion for RNA-seq data with DESeq2. Genome Biol.

[68] Leek JT, Johnson WE, Parker HS, Jaffe AE, Storey JD (2012). The sva package for removing batch effects and other unwanted variation in high-throughput experiments. Bioinformatics.

[69] McCarthy DJ, Campbell KR, Lun ATL, Wills QF (2017). Scater: pre-processing, quality control, normalization and visualization of single-cell RNA-seq data in R. Bioinformatics.

[70] Sergushichev AA. An algorithm for fast preranked gene set enrichment analysis using cumulative statistic calculation. Preprint at https://www.biorxiv.org/content/10.1101/060012v1.

[71] Jiang H, Lei R, Ding S-W, Zhu S (2014). Skewer: a fast and accurate adapter trimmer for next-generation sequencing paired-end reads. BMC Bioinformatics.

[72] Li H, Durbin R (2010). Fast and accurate long-read alignment with Burrows–Wheeler transform. Bioinformatics.

[73] Li H, Handsaker B, Wysoker A, Fennell T, Ruan J, Homer N (2009). The sequence alignment/map format and SAMtools. Bioinformatics.

[74] Quinlan AR, Hall IM (2010). BEDTools: a flexible suite of utilities for comparing genomic features. Bioinformatics.

[75] Zhang Y, Liu T, Meyer CA, Eeckhoute J, Johnson DS, Bernstein BE (2008). Model-based analysis of ChIP-Seq (MACS). Genome Biol.

[76] Heinz S, Benner C, Spann N, Bertolino E, Lin YC, Laslo P (2010). Simple combinations of lineage-determining transcription factors prime cis-regulatory elements required for macrophage and B cell identities. Mol Cell.

[77] Javierre BM, Burren OS, Wilder SP, Kreuzhuber R, Hill SM, Sewitz S (2016). Lineage-specific genome architecture links enhancers and non-coding disease variants to target gene promoters. Cell.

[78] Kumar S, Ambrosini G, Bucher P (2016). SNP2TFBS – a database of regulatory SNPs affecting predicted transcription factor binding site affinity. Nucleic Acids Res.

