# Supplementary material for: Genomic profiling of T-cell activation suggests increased sensitivity of memory T cells to CD28 costimulation
Source: Genes Immun. 2020 Nov 23;21(6):390–408. doi: 10.1038/s41435-020-00118-0 (PMC7785515; doi:10.1038/s41435-020-00118-0)
Supplement: Supplementary file 1 — Supplementary Tables and Figures legends [file 41435_2020_118_MOESM1_ESM.docx]

Supplementary Material

# Supplementary Table Legends S1-S4

**Table S1.** Differential expression analysis output tables from DESeq2 from the RNA data pairwise comparison between naive and memory cells in the resting and stimulated state. Only genes that are significant in at least one of the two analyses are included.

**Table S2.** Differential expression analysis output tables from DESeq2 from the RNA data pairwise comparison between stimulated states and the resting state.

**Table S3.** Differential expression analysis output tables from DESeq2 from the RNA data linear and switch models comparisons.

**Table S4.** List of switcher genes identified in the study.

# Supplementary Figure Legends S1-S6

**Supplementary Figure 1**. **A.** Gating strategy for T cell stimulation with DG75 and TSST-1 (corresponds to Figure 1A). **B.** Gating strategy for T cell stimulation with CHO-FcR (corresponds to Figure 1C). **C.** Gating strategy for T cell stimulation with DG75 and OKT3 (corresponds to Figure 1D). **D.** Level of CD86 expression on CD86GFP^+^ and CD86 knock-out (KO) DG75 cells. CD86 level was measured both using GFP (left histogram) and antibody staining (right histogram).

**Supplementary Figure 2**. **A.** Overview of study design. In brief, we isolated CD4 T cells from eight healthy individuals. Cells were cultured in seven different conditions, six of which included variable concentrations of anti-CD3 and anti-CD28 stimuli. In parallel, resting cells were cultured as a control. Cell culture constitutes a mix of cells that undergo activation and cell that remain in resting state because they did not encountered the stimulation or did not respond to it. To ensure we measure cellular response to successful stimulation, we generated sequencing data from sorted activated cells. **B**. Percentage of T cells expressing CD25 and CD69 three days after stimulation. The cells were stimulated using variable combinations of anti-CD3 antibody and CHO-CD86 cells. These were used to determine the concentrations of each stimulus in the RNA-seq experiment. **C.** Percentage of activated naive and memory T cells upon stimulation across the experimental conditions. Cells we sorted based on the CD4+CD127+CD45RA+ for naive and CD4+CD127+CD45RA- for memory T cells. Activation was measured based on CD25 expression. **D**. Percentage of the total variance explained by stimulation, cell type and blood processing batch, before and after batch correction.

**Supplementary Figure 3**. **A.** Number of TCR and CD28 sensitive genes identified by the linear (priority) and the switch model in the two cell types. **B.** Number of differentially expressed genes (DEG) upon stimulation. The coloring represents different stimulatory dependencies.

**Supplementary Figure 4**. Gating strategy for cytokine stainings (corresponds to Figure 4D).

**Supplementary Figure 5**. **A.** Number of peaks called per cell type and condition across the two donors. **B.** Enrichment score in open chromatin marked by ATAC and active promoters and enhancers marked by H3K27ac tested using all peaks per stimulation and cell and the resting state of each cell type as background. **C** Cytokine, chemokine and costimulatory molecules where the chromatin deposition around the gene (+- 150kb) is predictive of its sensitivity. Enrichment score was calculated as log2 coverage in the stimulated state divided by the coverage in the resting state. Shown are genes with enrichment score > 0.5. **D.** Selected example of the chromatin around *CD28* gene.

**Supplementary Figure 6**. Number of genes driving the enrichment with GWAS associated SNPs in peaks within a 150kb window and number of genes driving the enrichment with SNPs affecting a TF binding site motif in peaks within a 150kb window.
