# Supplementary figures and images for: Genomic profiling of T-cell activation suggests increased sensitivity of memory T cells to CD28 costimulation
Source: Genes Immun. 2020 Nov 23;21(6):390–408. doi: 10.1038/s41435-020-00118-0 (PMC7785515; doi:10.1038/s41435-020-00118-0)

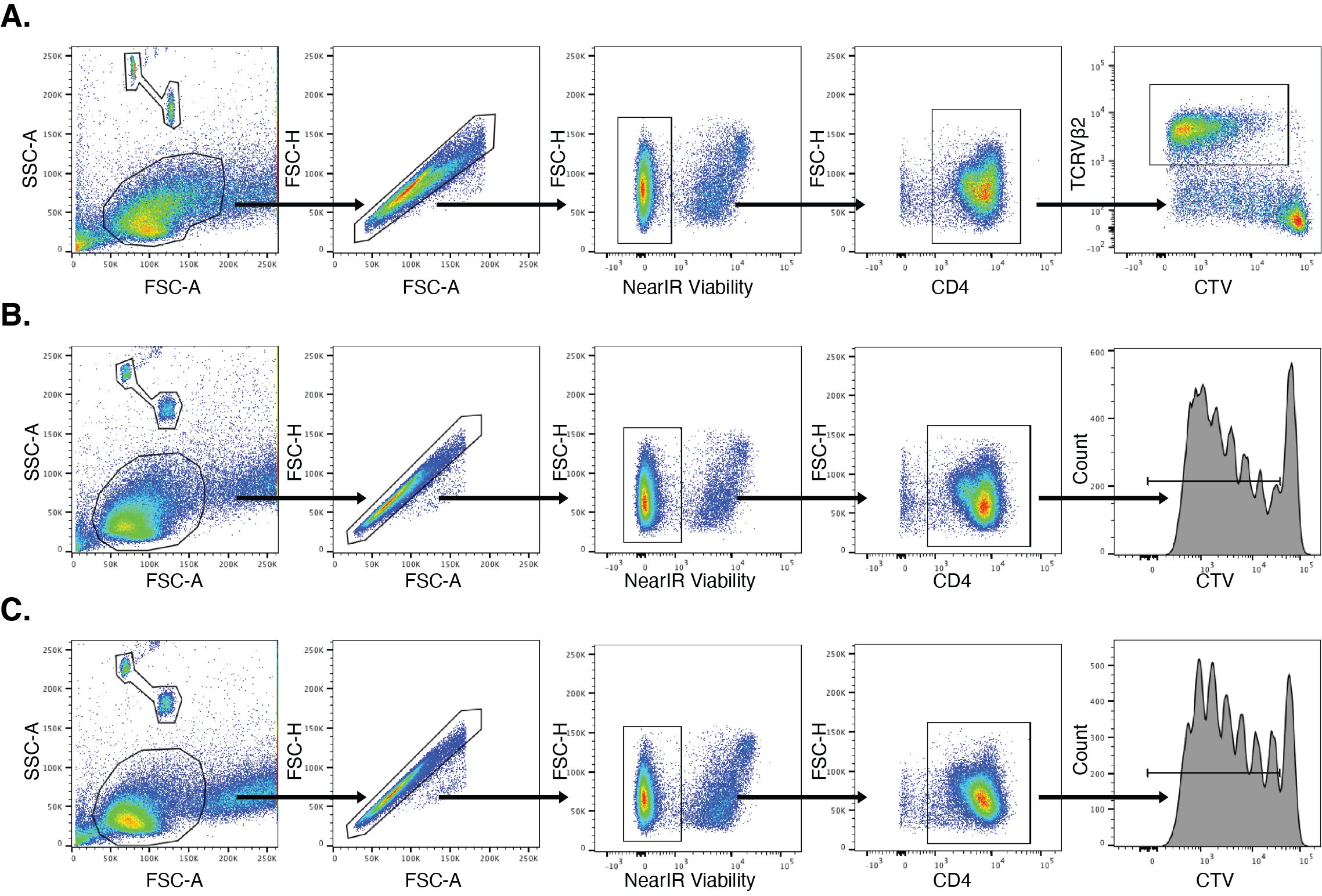

Supplement: Supplementary file 2 — Suppl. Figure 1 [file 41435_2020_118_MOESM2_ESM.tif]

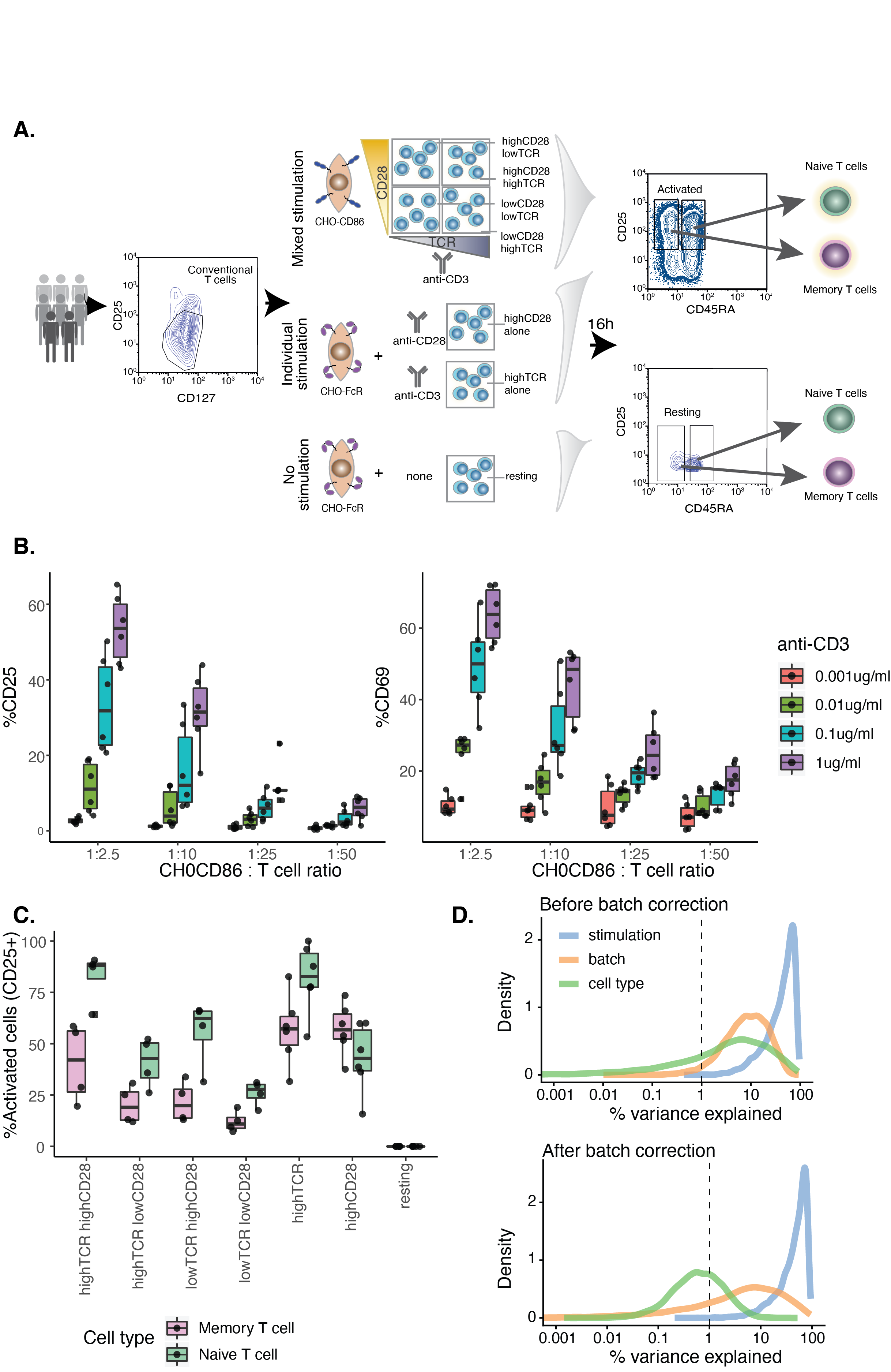

Supplement: Supplementary file 3 — Suppl. Figure 2 [file 41435_2020_118_MOESM3_ESM.tif]

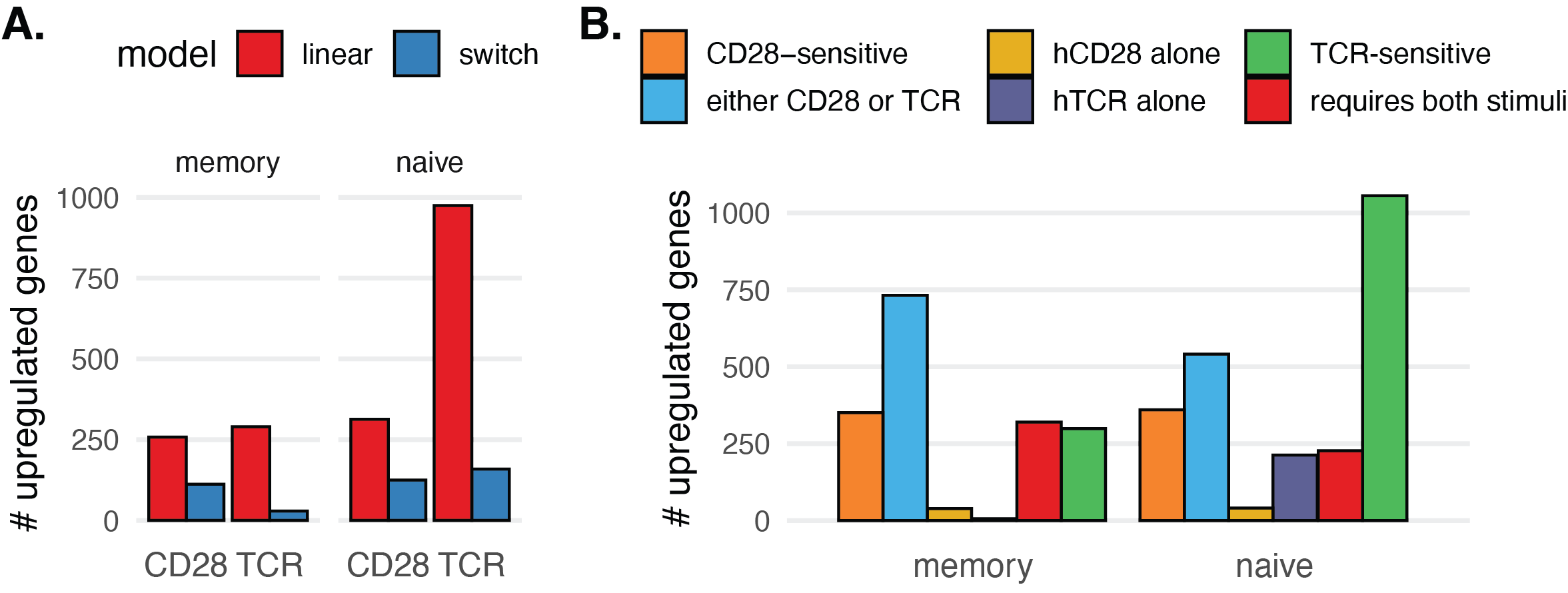

Supplement: Supplementary file 4 — Suppl. Figure 3 [file 41435_2020_118_MOESM4_ESM.tif]

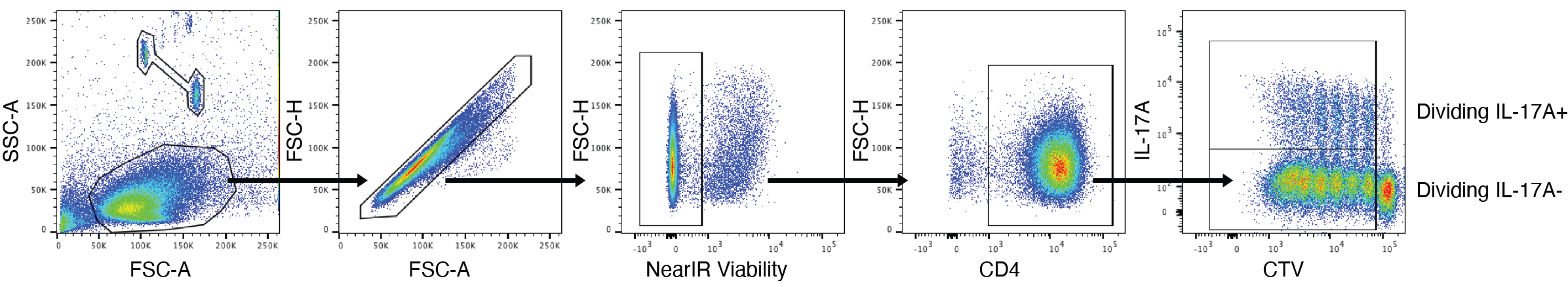

Supplement: Supplementary file 5 — Suppl. Figure 4 [file 41435_2020_118_MOESM5_ESM.tif]

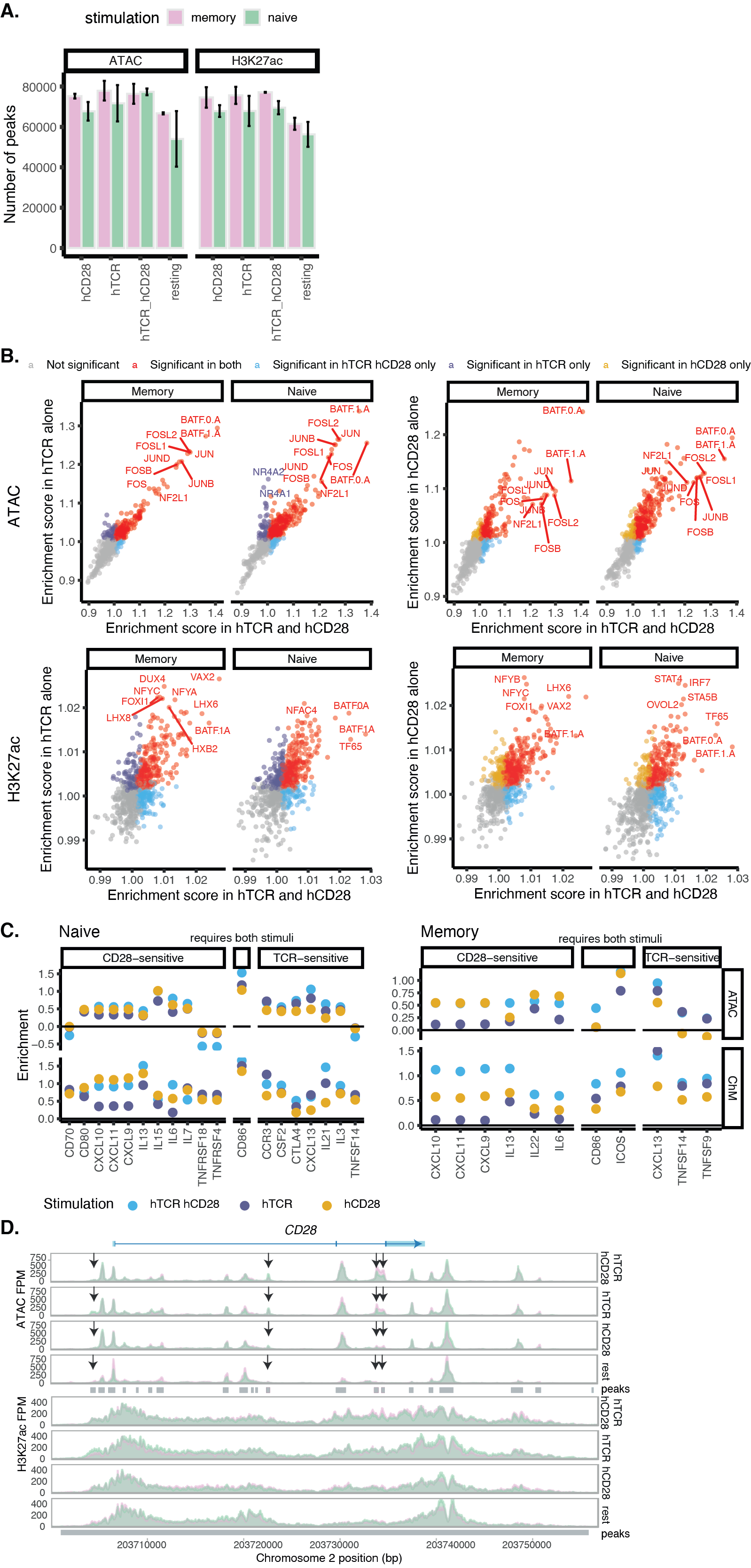

Supplement: Supplementary file 6 — Suppl. Figure 5 [file 41435_2020_118_MOESM6_ESM.tif]

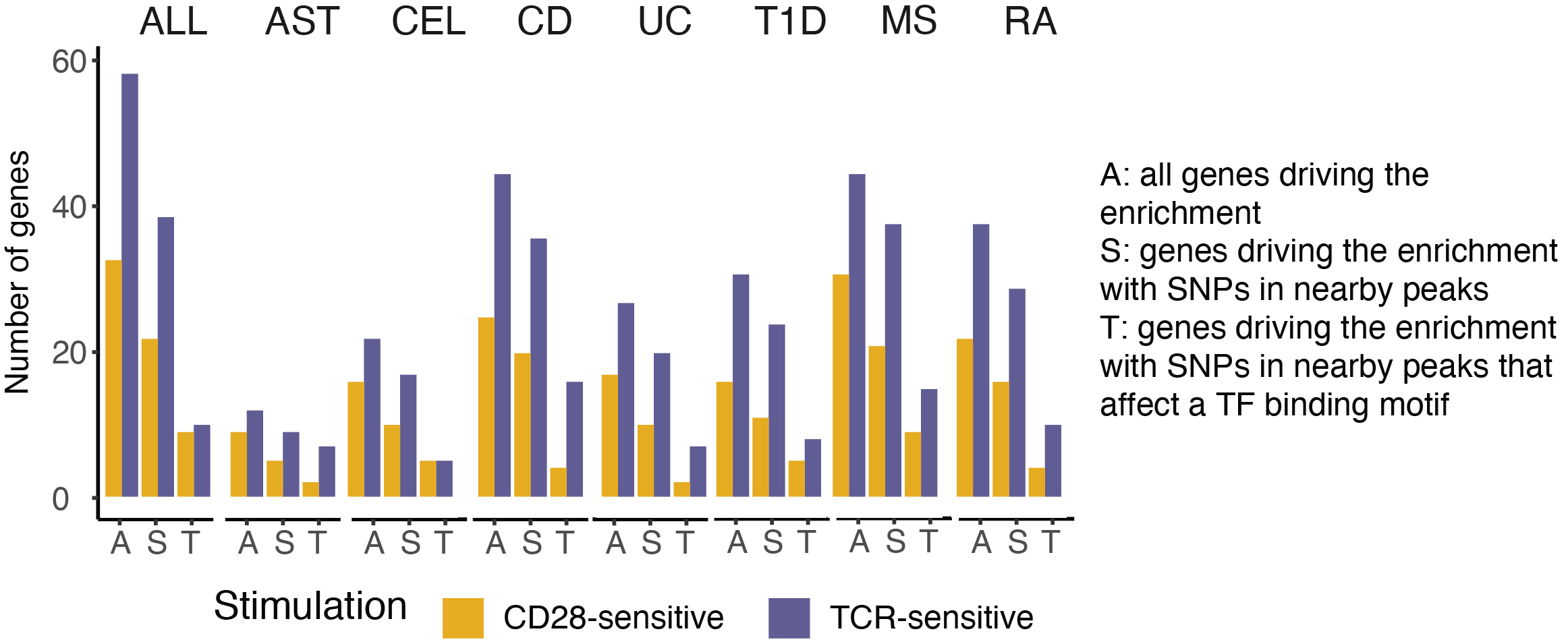

Supplement: Supplementary file 7 — Suppl. Figure 6 [file 41435_2020_118_MOESM7_ESM.tif]
